# Tumor‐Derived Exosomal circAP2B1 Induces M2 Macrophage Polarization by Enhancing Mitochondrial Homeostasis to Promote Esophageal Squamous Cell Carcinoma Progression

**DOI:** 10.1002/advs.76903

**Published:** 2026-07-31

**Authors:** Yiru Wang, Gang Feng, Youyu Wang, Ruihao Liang, Haixia Pan, Kai Lei

**Affiliations:** ^1^ Department of Oncology & Cancer Institute Sichuan Academy of Medical Sciences Sichuan Provincial People's Hospital University of Electronic Science and Technology of China Chengdu China; ^2^ Department of Thoracic Surgery Sichuan Academy of Medical Sciences Sichuan Provincial People's Hospital University of Electronic Science and Technology of China Chengdu China; ^3^ Department of Thoracic Surgery Sun Yat‐sen Memorial Hospital Sun Yat‐sen University Guangzhou China

**Keywords:** circAP2B1, esophageal squamous cell carcinoma, exosomes, mitochondrial homeostasis, tumor‐associated macrophages

## Abstract

Esophageal squamous cell carcinoma (ESCC) remodels the immunosuppressive tumor microenvironment via exosome‐mediated intercellular communication. In this study, circular RNA circAP2B1 is identified as a critical regulatory molecule that is markedly upregulated in ESCC tissues and patient serum and strongly associated with poor prognosis. Mechanistically, tumor‐derived exosomes efficiently deliver circAP2B1 to tumor‐associated macrophages (TAMs), where it serves as a distinct molecular scaffold that simultaneously binds the transcription factor ESRRA and the nuclear import receptor KPNA1, facilitating ternary complex formation and ESRRA nuclear translocation. Once in the nucleus, ESRRA directly activates the transcription of Mitofusin 2 (MFN2), a pivotal regulator of mitochondrial fusion, thereby enhancing mitochondrial oxidative phosphorylation, improving ATP production efficiency, and establishing a metabolically optimized intracellular environment that ultimately drives TAMs toward a pro‐tumor M2 phenotype. Both in vitro and in vivo experiments demonstrate that targeted intervention of the circAP2B1/ESRRA/KPNA1/MFN2 signaling axis effectively reverses M2 polarization and markedly suppresses tumor progression. This study uncovers a novel exosomal circRNA‐mediated metabolic‐immune regulatory pathway and offers new avenues for the diagnosis and treatment of ESCC. The findings not only expand the understanding of circRNA functions in tumor immunity but also provide a theoretical basis for the development of therapies targeting the metabolic‐immune axis.

## Graphical abstract

ESCC‐derived exosomal circAP2B1 promotes tumor progression by reprogramming mitochondrial metabolism via the ESRRA/KPNA1/MFN2 axis to induce M2 polarization of macrophages.

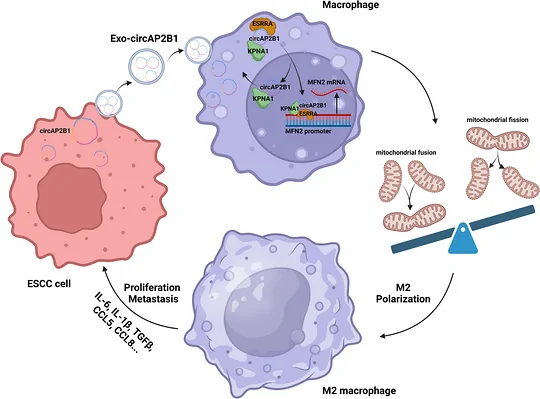

**Abbreviations**
ESCC: esophageal squamous cell carcinoma
TAMs: tumor‐associated macrophages
TME: tumor microenvironment
circRNA: circular RNA
scRNA‑seq: single‑cell RNA‑sequencing
ESRRA: estrogen‐related receptor alpha
KPNA1: Karyopherin Subunit Alpha 1
MFN2: Mitofusin 2
OXPHOS: oxidative phosphorylation
ROS: reactive oxygen species
ATP: adenosine triphosphate
FBS: fetal bovine serum
PMA: Phorbol 12‐Myristate 13‐Acetate
IL‐4: Interleukin‐4
RT‐qPCR: reverse transcription quantitative polymerase chain reaction
WB: western blot
TEM: transmission electron microscopy
IHC: immunohistochemistry
FISH: fluorescence in situ hybridization
IF: immunofluorescence
Co‐IP: co‐immunoprecipitation
RIP: RNA immunoprecipitation
ChIP: chromatin immunoprecipitation
CHIRP: chromatin isolation by RNA purification
SPR: surface plasmon resonance
TEM: transmission electron microscopy
NTA: nanoparticle tracking analysis
OCR: oxygen consumption rate
EMT: epithelial‐mesenchymal transition
OS: overall survival
GEO: gene expression omnibus
shRNA: short hairpin RNA
cDNA: complementary DNA
gDNA: genomic DNA

## 1 Introduction

Esophageal squamous cell carcinoma (ESCC) remains one of the most lethal malignancies worldwide, posing a substantial threat to human health owing to its highly aggressive behavior and poor prognosis [1, 2]. Despite advances in diagnosis and therapy, the molecular mechanisms underlying ESCC progression are not yet fully elucidated, highlighting the urgent need to identify novel therapeutic targets.

The tumor microenvironment (TME) plays a central role in ESCC progression. Within the TME, tumor‐associated macrophages (TAMs) polarized toward the M2 phenotype promote tumor growth and metastasis through multiple mechanisms, including immunosuppression, angiogenesis, and stromal remodeling [3, 4, 5]. Consequently, identifying key factors within the TME that regulate TAM polarization and elucidating their underlying mechanisms are critical for developing therapeutic strategies aimed at reversing the immunosuppressive TME and inhibiting tumor progression. Emerging evidence indicates that exosomal circRNA‐mediated crosstalk between tumor cells and TAMs represents a pivotal molecular event sustaining the high invasive and metastatic potential of ESCC [6, 7]. Moreover, metabolic reprogramming, particularly the regulation of mitochondrial function in TAMs, is closely associated with their polarization phenotype [8, 9]. These observations prompted an investigation into the role of ESCC‐derived exosomes in modulating mitochondrial homeostasis in TAMs.

Exosomes function as critical mediators of intercellular communication by delivering functional molecules, including non‐coding RNAs, to remodel the TME [10, 11]. Among these molecules, circular RNAs (circRNAs) have emerged as pivotal regulators within cancer networks due to their pronounced stability and tissue‐specific expression [12]. Previous studies have demonstrated that dysregulated circRNAs in ESCC contribute to tumor proliferation, metastasis, and stemness by acting as microRNA (miRNA) sponges [13] or via cap‐independent translation [14, 15]. Recent evidence further indicates that exosomal circRNAs can modulate immune cell function, thereby influencing tumor progression [16, 17]. Nevertheless, the specific role and underlying mechanism of exosomal circRNAs in mediating TAM polarization in ESCC remain largely unexplored.

This study investigates the regulatory function of ESCC cell‐derived exosomal circRNAs in TAM polarization. CircRNA microarray analysis revealed, for the first time, that tumor‐derived exosomal circAP2B1 is markedly upregulated in both tumor tissues and serum from patients with ESCC, with its expression correlating with tumor progression and poor prognosis. Subsequent experiments demonstrated that ESCC‐derived circAP2B1 can be delivered into TAMs via exosomes, where it facilitates the assembly of a circAP2B1/ESRRA/KPNA1 ternary complex. This complex promotes the nuclear translocation of the transcription factor estrogen‐related receptor α (ESRRA), subsequently activating Mitofusin 2 (MFN2) transcription to enhance mitochondrial homeostasis and drive M2 polarization. Both in vitro and in vivo assays confirmed that this axis substantially promotes ESCC proliferation, metastasis, and epithelial‐mesenchymal transition (EMT).

The novelty of this study lies in elucidating a previously unrecognized mechanism by which tumor‐derived exosomal circAP2B1 orchestrates M2 polarization of TAMs through metabolic reprogramming of mitochondrial homeostasis. These findings not only suggest a potential diagnostic biomarker and therapeutic target for ESCC but also broaden the understanding of circRNA function within the tumor immune microenvironment, providing a theoretical basis for immune therapies targeting mitochondrial metabolism.

## 2 Methods

### 2.1 Cell Lines and Culture

Human embryonic kidney 293T cells, human normal esophageal epithelial cells (HEEC), human ESCC cell lines (KYSE30, KYSE450, ECA109, and TE1), human monocytic cells (THP‐1), and mouse monocytic leukemia cells (RAW264.7) were obtained from the Cell Bank of the Chinese Academy of Sciences (Shanghai, China). 293T, HEEC, and ESCC cell lines were maintained in Dulbecco's Modified Eagle Medium (DMEM; Gibco, USA), whereas THP‐1 and RAW264.7 cells were cultured in RPMI‐1640 medium (Gibco). All media were supplemented with 10% fetal bovine serum (FBS; Gibco) and 1% penicillin‐streptomycin (Biosharp, China). For the co‐culture model, THP‐1 cells stimulated with phorbol 12‐myristate 13‐acetate (PMA; 0.4 µmol/L) were seeded in the lower chamber, while ESCC cells were seeded in the upper chamber. All cells were incubated at 37 °C in a humidified atmosphere containing 5% CO_2_.

### 2.2 Induction of M2 Macrophage Polarization by IL‐4 Stimulation

THP‐1 cells were initially stimulated with 100 ng/mL PMA for 24 h to induce differentiation into M0 macrophages. Subsequently, the cells were treated with 50 ng/mL interleukin‐4 (IL‐4) for an additional 24 h to promote M2 polarization. Analyses were conducted 48 h after the initial stimulation.

### 2.3 Human Samples and circRNA Microarray Analysis

Tumor tissues, adjacent normal esophageal epithelial tissues, and serum samples were collected from a cohort of 158 patients with ESCC who underwent surgical resection at the Department of Thoracic Surgery, Sichuan Provincial People's Hospital between 2015 and 2019. Control serum samples (n  =  158) were obtained from healthy volunteers at the same institution. These specimens were employed for subsequent experiments, including exosome isolation, reverse transcription‐quantitative polymerase chain reaction (RT‐qPCR), fluorescence in situ hybridization (FISH), immunohistochemistry (IHC), and Western blotting (WB). The study involving human subjects was conducted in accordance with the principles of the Declaration of Helsinki and received approval from the Ethics Committee of Sichuan Provincial People's Hospital (Approval No: 2025–227).

CircRNA expression profiling was performed using the Arraystar Human circRNA Microarray (Aksomics, China). Differential expression analyses were conducted on three pairs of ESCC tissues and matched adjacent normal tissues, as well as on serum samples from three patients with ESCC and three healthy controls. All raw microarray data have been deposited in the Gene Expression Omnibus (GEO) database under accession numbers GSE250413 (tissues) and GSE296251 (serum).

### 2.4 Single‑cell RNA‑Sequencing (scRNA‑seq) Analysis

Fresh ESCC tissues (three pairs of circAP2B1‐high and circAP2B1‐low samples) were minced and enzymatically dissociated using a human tumor dissociation kit (Miltenyi Biotec) on a gentleMACS tissue processor. The resulting cell suspension was filtered sequentially through 70 and 40 µm strainers, and cell viability (>85%) was assessed by trypan blue exclusion. Single‐cell libraries were prepared using the Chromium Next GEM Single‐Cell 3' Kit v3.1 (10 × Genomics) according to the manufacturer's protocol and sequenced on a NovaSeq 6000 platform (Illumina) in paired‐end mode (150 bp) with a target depth of ≥ 50 000 cells per sample. Raw sequencing data were processed using Cell Ranger (v7.0.1) and aligned to the human reference genome GRCh38 to generate gene–cell expression matrices. Quality control was performed using Seurat (v4.3.0); cells with 200–5000 detected genes and < 20% mitochondrial reads were retained, and ambient RNA and doublets were removed using SoupX and DoubletFinder, respectively. After log‐normalization, 2,000 highly variable genes were selected for principal component analysis (PCA). Clustering was conducted using the Louvain algorithm (resolution 0.5) based on the first 15 principal components, and results were visualized via UMAP/t‐SNE. Cell types were annotated by integrating automated annotation using SingleR with manual validation based on known marker genes (EPCAM, CD68, CD3D, NKG7, etc.). The enrichment score of M2 signature gene sets (MsigDB, including ARG1, CD163, IL10, etc.) was calculated for each macrophage using the ssGSEA algorithm implemented in the GSVA package, and M2 scores were compared between circAP2B1‐high and circAP2B1‐low groups using the Wilcoxon test. Percentages of different immune cell subsets were computed for each sample, and intergroup comparisons were performed using Student's t‐test or the Mann–Whitney U test, as appropriate.

### 2.5 Animal Experiments

All animal procedures were approved by the Institutional Animal Care and Use Committees of Sichuan Provincial People's Hospital and the University of Electronic Science and Technology of China (Approval No: 2025–227). Female BALB/c nude mice (4–6 weeks old) were maintained under specific pathogen‐free conditions.

Exosomes were isolated from the culture supernatant of KYSE30 cells stably overexpressing circAP2B1 and incubated with PMA‐induced mature macrophages for 48 h. These pretreated macrophages were subsequently co‐cultured with KYSE30 cells stably transfected with a luciferase reporter.

For the subcutaneous xenograft model, a total of 5 × 10^6^ co‐cultured cells (macrophages + KYSE30‐luciferase) were injected subcutaneously into the left flank of each mouse (n  =  6 per group). The macrophage‐to‐KYSE30 cell ratio was 1:1, corresponding to 2.5 × 10^6^ cells of each type. Tumor volume was measured weekly and calculated using the formula: V = 1/2 × L × W^2^, where L and W represent the longest and shortest tumor diameters, respectively. Mice were euthanized six weeks post‐injection. Subcutaneous tumors were excised, weighed, and collected for further analysis. Hematoxylin and eosin (H&E) staining and IHC were performed to assess the expression of Ki‐67 (proliferation marker), E‐cadherin and N‐cadherin (EMT markers), and MFN2.

For the lung metastasis model, 2 × 10^6^ co‐cultured cells were injected via the tail vein of each mouse (n  =  6 per group). The ratio of macrophages to KYSE30 tumor cells was maintained at 1:1 (1 × 10^6^ macrophages and 1 × 10^6^ KYSE30 cells). Six weeks later, mice were anesthetized and imaged using the Tanon ABL X6 In Vivo Imaging System (Tanon, China). Lung tissues were collected for H&E staining, and the number of metastatic nodules was quantified.

### 2.6 Plasmid Construction, Lentivirus Production, and Cell Transfection

Plasmids for circAP2B1 overexpression, sh‐circAP2B1, sh‐ESRRA, sh‐KPNA1, and sh‐MFN2 were constructed and packaged into lentiviruses by GeneChem (China). ESCC cells or macrophages were transduced with concentrated viruses using LipoFilter transfection reagent (Hanbio, China). Stable cell lines were selected and maintained with 2 µg/mL puromycin. The sequences of all shRNAs are provided in Table S1.

### 2.7 Reverse Transcription‐Quantitative Polymerase Chain Reaction (RT‐qPCR)

Total RNA was extracted from cells or tissues using TRIzol reagent (Life Technologies, USA). According to the manufacturer's instructions, 1,000 ng of total RNA was reverse‐transcribed into cDNA using HiScript II Q RT SuperMix (Vazyme, China). RT‐qPCR was conducted using AceQ qPCR SYBR Green Master Mix (Vazyme) on a LightCycler 96 System (Roche, Switzerland). β‐actin (ACTB) was used as an internal control. Relative gene expression was calculated using the 2^−ΔΔCT^ method. Primer sequences are detailed in Table S2.

### 2.8 Western Blotting (WB)

Cells or tissue samples were lysed in RIPA buffer supplemented with 1% protease inhibitor cocktail. Protein concentrations were determined using a BCA assay. Equal amounts of protein (20–50 µg) were separated by SDS‐PAGE and transferred onto PVDF membranes. Membranes were blocked with 5% non‐fat milk for 1 h at room temperature, followed by incubation with primary antibodies overnight at 4 °C. After washing with TBST, membranes were incubated with HRP‐conjugated secondary antibodies for 1 h at room temperature. Protein bands were visualized using an ECL substrate kit (Vazyme) and quantified with ImageJ software. Antibody details are provided in Table S3. All experiments were independently repeated three times, yielding consistent results.

### 2.9 Transmission Electron Microscopy (TEM)

For quantitative TEM analysis, images were obtained from three independent biological replicates. For each condition, at least 30 randomly selected cells were examined, and a minimum of 10 well‐sectioned mitochondria per cell were analyzed. Mitochondrial area (µm^2^) and aspect ratio (length/width) were quantified using ImageJ software. Cristae number per mitochondrion was manually counted by two independent researchers blinded to the experimental groups.

### 2.10 Immunohistochemistry (IHC)

Tissue samples were fixed in 10% neutral‐buffered formalin for 24 h, embedded in paraffin, and sectioned. Following deparaffinization and rehydration, antigen retrieval was performed using citrate buffer under heat‐mediated conditions. Endogenous peroxidase activity was inhibited with 3% H_2_O_2_, and non‐specific binding was blocked with 5% bovine serum albumin (BSA). Sections were incubated with primary antibodies overnight at 4°C, followed by incubation with horseradish peroxidase (HRP)‐conjugated secondary antibodies for 1 h at room temperature. Color development was carried out using 3,3'‐diaminobenzidine (DAB), and nuclei were counterstained with hematoxylin. Detailed antibody information is provided in Table S3.

### 2.11 Chromatin Immunoprecipitation (ChIP)

ChIP assays were conducted using the High‐Sensitivity ChIP Kit (#ab185913, Abcam, UK) in accordance with the manufacturer's instructions. Cells were cross‐linked with 1% formaldehyde, and the reaction was quenched with glycine. Chromatin was sheared by sonication into fragments of 200–500 bp. Lysates were pre‐cleared with Protein A/G magnetic beads and subsequently incubated with target‐specific antibodies or control IgG overnight at 4°C. Immune complexes were captured with beads and washed sequentially with low‐salt, high‐salt, lithium chloride (LiCl), and TE buffers. DNA was then eluted, reverse cross‐linked, purified, and analyzed by PCR using primers specific to the target genomic regions. Data were normalized to input DNA and IgG controls. All experiments were independently repeated three times, yielding consistent results.

### 2.12 Chromatin Isolation by RNA Purification (ChIRP)

The ChIRP assay was performed using the Magna ChIRP RNA Interactome Kit (#17‐10494, Sigma, Germany) according to the manufacturer's protocol. Cells were cross‐linked with 1% formaldehyde, and the reaction was quenched with glycine. Chromatin was fragmented by sonication to 200–500 bp. Biotin‐labeled DNA probes targeting circAP2B1 were hybridized with the lysates overnight at 42°C. Probe‐RNA complexes were captured using streptavidin magnetic beads and washed sequentially with low‐salt, high‐salt, and stringent wash buffers. Bound DNA/RNA was digested with proteinase K and subsequently purified. Enrichment of target DNA was assessed by PCR or qPCR using primers specific to the RNA‐associated genomic regions. Data were normalized to input DNA and negative control probes. All experiments were independently repeated three times, yielding consistent results.

### 2.13 RNA Pull‐Down

The RNA pull‐down assay was conducted using the Pierce Magnetic RNA‐Protein Pull‐Down Kit (#21115, Thermo Scientific, USA) following the manufacturer's instructions. Biotinylated circAP2B1‐specific and negative control probes were designed and synthesized by GenePharma (China). Probes (30 µg) were incubated with 40 µL of streptavidin magnetic beads for 30 min at room temperature. Cells were lysed in RIP buffer supplemented with RNase and protease inhibitors. Cell lysates were incubated with the probe‐bead complexes for 4 h at 4°C with rotation. Following washes with RIP wash buffer, bound complexes were eluted and digested with proteinase K buffer at 55°C for 30 min with shaking. Recovered RNA or proteins were analyzed by RT‐PCR, WB, or mass spectrometry. All experiments were independently repeated three times, yielding consistent results.

### 2.14 RNA Immunoprecipitation (RIP)

The RIP assay was performed using the BeyoRIP RIP Assay Kit (#P1801S, Beyotime, China) according to the manufacturer's instructions. Cell lysates containing RNase inhibitors were incubated with target protein‐specific antibodies or control IgG overnight at 4°C. Antibody‐RNA complexes were captured using Protein A/G magnetic beads and washed sequentially with high‐salt buffer (containing 0.1% SDS) and RIP wash buffer. Bound RNA was released by proteinase K digestion, extracted with TRIzol, and analyzed by qPCR. All experiments were independently repeated three times, yielding consistent results.

### 2.15 Co‐Immunoprecipitation (Co‐IP)

Co‐IP assays were carried out using the PureBinding Co‐Immunoprecipitation Kit (#P0401, GENESEED, China) in accordance with the manufacturer's instructions. Cell lysates containing protease inhibitors were incubated with target‐specific antibodies or control IgG overnight at 4°C. Antibody‐protein complexes were captured with Protein A/G magnetic beads and washed sequentially with low‐salt (150 mM NaCl), high‐salt (500 mM NaCl), and neutral wash buffers. Bound proteins were eluted by boiling in SDS loading buffer and detected by WB. All experiments were independently repeated three times, yielding consistent results.

### 2.16 Fluorescence In Situ Hybridization (FISH)

Subcellular localization of circAP2B1 was determined using an RNA FISH kit (#C10910, RiboBio, China) following the manufacturer's protocol. A 5'‐Cy3‐labeled probe specifically targeting the circAP2B1 backsplice junction was used. Images were acquired using a Zeiss LSM 710 laser scanning confocal microscope (Zeiss, Germany). The FISH probe sequence for circAP2B1 was Cy3‐GAAAGAGAGAACATTGGATTTG.

### 2.17 Multiplex Immunofluorescence (mIF)

Cells on coverslips were fixed with 4% paraformaldehyde for 15 min and permeabilized with 0.1% Triton X‐100 for 10 min. Staining was performed using the Treble‐Fluorescence Immunohistochemical Mouse/Rabbit Kit (#RS0035, Immunoway, USA) in accordance with the manufacturer's instructions. Endogenous peroxidase activity was blocked, followed by incubation with primary antibodies diluted in PBS overnight at 4°C. After washing, HRP‐conjugated secondary antibodies were applied. Tyramide signal amplification was sequentially performed using Alexa Fluor 488, 594, and 647, with microwave‐assisted antibody stripping between each round. Nuclei were counterstained with 4',6‐diamidino‐2‐phenylindole (DAPI). Images were captured using a spectral confocal microscope with the following filters: DAPI (345/455 nm), FITC (496/519 nm), TRITC (590/617 nm), and Cy5 (650/665 nm).

### 2.18 Surface Plasmon Resonance (SPR) Analysis

SPR experiments were conducted on a Biacore T200 instrument (Cytiva) at 25 °C. Biotinylated circAP2B1 was immobilized onto a streptavidin (SA) sensor chip at approximately 200 response units (RU). The ESRRA/KPNA1 complex was pre‐formed by mixing recombinant proteins at an equimolar ratio and serially diluted to 7–224 µM in HBS‐EP+ buffer containing 0.1 U/µL RNaseOUT. Binding interactions were measured at a flow rate of 30 µL/min, with 60 s for association and 60 s for dissociation. Steady‐state binding data were fitted to a 1:1 Langmuir model using Biacore T200 Evaluation Software. Each concentration was tested in triplicate, and the experiment was independently repeated three times.

### 2.19 Measurement of Mitochondrial Oxygen Consumption Rate (OCR)

Mitochondrial OCR was measured using a Seahorse XF24 Analyzer (Seahorse Bioscience, USA) according to the manufacturer's instructions and previously published methods. Macrophages (5 × 10^4^ cells/well) were seeded into XF24 microplates (#101122‐100). The sensor cartridge was hydrated overnight in XF calibrant solution at 37°C. Prior to the assay, cells were washed and equilibrated for 1 h in XF base DMEM medium (pH 7.4) supplemented with 10 mM glucose, 1 mM sodium pyruvate, and 2 mM L‐glutamine at 37°C in a non‐CO_2_ incubator. Sequential injections of 1.0 µM oligomycin, 1.0 µM FCCP, and 0.5 µM rotenone/antimycin A (Rot/AA) were performed using the XF Mitochondrial Stress Test Kit (#103010‐100). OCR was recorded in real time and analyzed using Wave software.

### 2.20 Intracellular ATP Level

Intracellular ATP levels were determined using an ATP assay kit (#BC0300, Solabio, China) according to the manufacturer's protocol. Briefly, 1 × 10^4^ cells were collected and lysed in 1 mL of extraction buffer, followed by sonication on ice. Lysates were centrifuged at 10 000 × g for 10 min at 4°C, and ATP content in the supernatant was measured and normalized to total protein concentration.

### 2.21 Exosome Isolation and Characterization

Exosomes were isolated from serum and cell culture supernatants using the Total Exosome Isolation Reagent (#4478359/4478360, Thermo Fisher Scientific, USA) according to the manufacturer's instructions, and their concentration was quantified by bicinchoninic acid (BCA) assay. Exosome morphology was visualized by TEM (JEM‐1400, JEOL, Japan). Exosomal markers were confirmed by WB, and nanoparticle tracking analysis (NTA) was performed by Bioyard Biotechnology Development Co., Ltd (China) to determine particle size distribution and concentration.

### 2.22 Dual‐Luciferase Reporter Assay

HEK293T cells were co‐transfected with firefly luciferase reporter plasmids (pGL4‐luc) containing wild‐type or mutant ESRRA promoter/3'UTR sequences and the Renilla luciferase control plasmid (pRL‐TK) at a 50:1 mass ratio using Lipofectamine 3000. Cells were lysed 48 h post‐transfection, and luciferase activities were measured sequentially using the Dual‐Luciferase Reporter Assay System (#E1910, Promega, USA). Firefly luciferase activity was normalized to Renilla luciferase activity for each sample.

### 2.23 Flow Cytometry Analysis

M2‐type macrophages were characterized by flow cytometry using antibodies against CD11b and CD206. Apoptosis in ESCC cells was assessed using an Annexin V‐FITC/PI Apoptosis Detection Kit (#C1062S, Beyotime). Intracellular reactive oxygen species (ROS) levels in macrophages were measured using a DCFH‐DA‐based ROS Assay Kit (#E‐BC‐K138‐F, Elabscience, China). Mitochondrial membrane potential was evaluated using a JC‐1 Assay Kit (#E‐CK‐A301, Elabscience, China). Flow cytometry data were acquired and analyzed using appropriate software.

### 2.24 Statistical Analysis

Statistical analyses were performed using SPSS Statistics v26.0 (IBM Corporation, USA) and GraphPad Prism v9.5 (GraphPad Software, USA). Single‐cell sequencing data were analyzed using RStudio. Categorical variables are presented as numbers (percentages) and compared using the Chi‐square test or Fisher's exact test, as appropriate. Continuous variables are described as mean ± standard deviation or median (interquartile range) and compared using the Student's t‐test or Mann‐Whitney U test, depending on data distribution. Survival analyses were conducted using the Kaplan‐Meier method and compared by the log‐rank test. Univariate and multivariate Cox proportional hazards models were applied to identify independent prognostic factors. Two‐sided *P*‐values < 0.05 were considered statistically significant.

### 2.25 Ethics Approval and Consent to Participate

The collection of all sample tissues has been approved by the Ethics Supervision Committee of Sichuan Provincial People's Hospital (approval number: 2025–227). All subjects in this study have signed informed consent forms. Animal experiments have also been approved by the Ethics Supervision Committee of Sichuan Provincial People's Hospital (approval number: 2025–227).

## 3 Results

### 3.1 circRNA Expression Profile in ESCC and Characterization of circAP2B1

To identify potential circRNAs associated with ESCC progression, circRNA microarray analyses were conducted on tumor tissues and paired adjacent normal tissues (NATs) from three patients with ESCC, as well as on serum samples from three patients with ESCC and three healthy controls. Distinct dysregulated circRNA expression profiles were obtained. Compared with controls, 296 circRNAs were upregulated in ESCC tissues (GSE250413), and 308 circRNAs were upregulated in patient serum (Figure 1A, B). Four circRNAs—circAP2B1, circCEP95, circGIT2, and circSNX14—were consistently upregulated in both ESCC tissues and serum (Figure 1C). Notably, RT‐qPCR analysis in an expanded cohort of 158 patients with ESCC confirmed that only circAP2B1 was significantly overexpressed upon further validation (Figure 1D, E). CircAP2B1 (hsa_circ_0004745), with a length of 382 nucleotides, is generated by back‐splicing of exons 4 and 5 of the AP2B1 gene, which was verified by Sanger sequencing of the back‐splice junction (Figure 1F). PCR amplification using divergent and convergent primers followed by agarose gel electrophoresis detected the back‐spliced form of circAP2B1, whereas the linear AP2B1 transcript was not observed (Figure 1G). CircAP2B1 exhibited greater stability than its linear counterpart, as evidenced by resistance to RNase R digestion and an extended half‐life in actinomycin D assays (Figure 1H, I). Furthermore, RNA nuclear/cytoplasmic fractionation (Figure 1J) and FISH (Figure 1K) revealed that circAP2B1 was predominantly localized in the cytoplasm of KYSE30 and TE1 cells.

**FIGURE 1:**
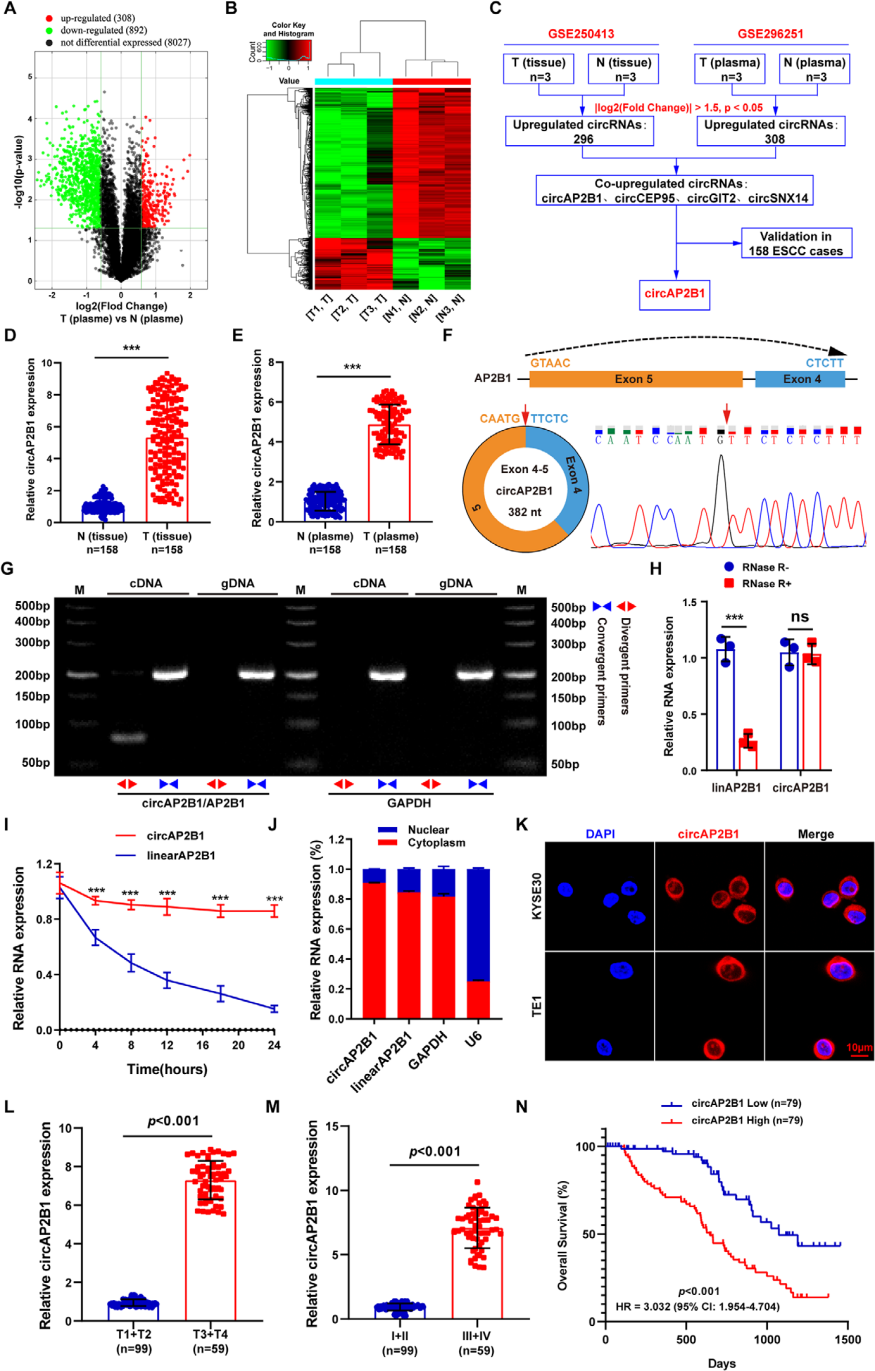
circRNA Expression Profile in ESCC and Characterization of circAP2B1. (A) Volcano plot of circRNA expression from microarray analysis of serum from 3 ESCC patients and 3 healthy controls. (B) Heatmap of circRNA expression from the same serum microarray analysis. (C) Flowchart for screening circAP2B1 as a candidate consistently upregulated in both ESCC tissues and serum. (D) RT‐qPCR analysis of circAP2B1 expression in 158 paired ESCC tissues and NATs. (E) ddPCR analysis of circAP2B1 expression in serum from 158 ESCC patients and 158 healthy controls. (F) Schematic diagram of the genomic locus of AP2B1 and the back‐splicing forming circAP2B1, confirmed by Sanger sequencing. (G) PCR analysis of circAP2B1 (divergent primers) and linear AP2B1 mRNA (convergent primers) in cDNA and gDNA from ESCC cells. (H, I) RNase R resistance assay (H) and Actinomycin D assay (I) evaluating the stability of circAP2B1 compared to linear AP2B1 mRNA. (J) Nuclear/cytoplasmic fractionation followed by qPCR analysis of circAP2B1 and linear AP2B1 mRNA localization in ESCC cells. GAPDH and U6 served as cytoplasmic and nuclear controls, respectively. (K) FISH assay determining the subcellular localization of circAP2B1 in ESCC cells. Scale bar = 10 µm. (L, M) RT‐qPCR analysis of exosomal circAP2B1 (Exo‐circAP2B1) expression in ESCC tissues stratified by N stage (L) and TNM stage (M). (N) Kaplan‐Meier analysis of overall survival (OS) in ESCC patients based on circAP2B1 expression levels. **p < 0.01, ***p < 0.001 and ns, not significant.

Clinically, elevated circAP2B1 expression was associated with more advanced pathological stages in patients with ESCC (Figure 1L, M; Table 1). Survival analysis demonstrated that patients with high circAP2B1 expression exhibited significantly poorer overall survival compared with those displaying low expression levels (Figure 1N). Moreover, univariate and multivariate Cox regression analyses identified high circAP2B1 expression as an independent risk factor for poor prognosis in patients with ESCC (Table 2).

**TABLE 1:** Relationship between general clinical characteristics and circAP2B1 level in patients with ESCC (n = 158).

General clinical characteristics	CircAP2B1 expression		p‐value	Statistics
	Low (n = 79)	High (n = 79)		
Gender (%)			1	0.025
Female	42 (53.2)	41 (51.9)		
Male	37 (46.8)	38 (48.1)		
Age (mean ± SD)	59.75 ± 4.068	57.8 ± 8.512	0.068	1.837
BMI (mean ± SD)	22.713 ± 3.464	22.834 ± 3.949	0.773	−0.289
Smoke history (%)	44 (55.7)	52 (65.8)	0.254	1.699
Alcohol history (%)	51 (64.6)	54 (68.4)	0.736	0.256
T stage (%)			0***	37.032
T1 + T2	68 (86.1)	31 (39.2)		
T3 + T4	11 (13.9)	48 (60.8)		
N stage (%)			0.094	3.375
N0	57 (72.2)	46 (58.2)		
N1 + N2 + N3	22 (27.8)	33 (41.8)		
TNM stage (%)			0.021*	6.086
I + II	37 (46.8)	22 (27.8)		
III + IV	42 (53.2)	57 (72.2)		

BMI Body Mass Index, a Fisher exact probability test, * *p* < 0.05, *** *p* < 0.001

**TABLE 2:** Univariate and multivariate Cox‐regression analysis of prognostic factors for patients with ESCC (n = 158).

Feature	n	Univariate analysis			Multivariate analysis		
		HR	95% CI	*p*	HR	95% CI	*p*
Gender							
Female	75	1					
Male	83	2.378	0.823–9.784	0.231			
Age							
<65	79	1					
≥65	79	1.067	0.713–1.597	0.065			
BMI							
<18.5	13	1					
≥18.5	145	0.726	0.364–1.450	0.365			
Smoking history							
No	62	1					
Yes	96	0.967	0.637–1.468	0.875			
Alcohol history							
No	53	1					
Yes	105	1.294	0.842–1.991	0.240			
T stage							
T1+T2	99	1					
T3+T4	59	2.671	1.722–4.025	0***	1.390	0.711‐2.720	0.366
N stage							
N0	103	1					
N1+N2+N3	55	2.006	1.342–2.997	0.001**	0.644	0.229‐1.811	0.404
TNM stage							
I+II	99	1					
III+IV	55	2.223	1.484–3.331	0***	0.188	0.692‐6.566	0.188
Expression of circAP2B1							
Low	79	1					
High	79	2.736	1.761–4.253	0***	1.943	1.108‐3.408	0.020*

n sample number, HR Hazard ratio, CI Confidence interval, BMI Body Mass Index * *p* < 0.05, ** p < 0.01, *** *p* < 0.001

To assess the diagnostic potential of serum exosomal circAP2B1, receiver operating characteristic (ROC) curve analysis was performed in the same cohort. Serum circAP2B1 demonstrated an area under the curve (AUC) of 0.7631 (95% confidence interval [CI]: 0.7118–0.8144), with 84.81% sensitivity and 51.90% specificity at a cut‐off value of 4.125 (Figure S1A). Compared with the conventional biomarker CEA (AUC = 0.6636), circAP2B1 exhibited superior diagnostic performance, and their combination further improved the AUC to 0.8723 (95% CI: 0.8359–0.9087). Additionally, high circAP2B1 expression was associated with significantly shorter progression‐free survival (log‐rank *p* < 0.001) (Figure S1B). These findings support circAP2B1 as a promising diagnostic and prognostic biomarker for ESCC.

### 3.2 circAP2B1 Overexpression Correlates With M2‐TAM Infiltration and Promotes ESCC Progression

To investigate the potential biological role of circAP2B1 in ESCC progression, FISH was performed on ESCC tissue sections. CircAP2B1 expression was markedly higher in ESCC tissues compared with NATs and increased progressively with advancing pathological stage (Figure 2A). To examine the relationship between TAM infiltration and ESCC progression, immunostaining for M2‐TAM markers (CD68, CD163, CD206) was conducted. M2‐TAM abundance was significantly elevated in ESCC tissues relative to NATs and exhibited stage‐dependent increases (Figure 2B). Analysis of the GEO dataset GSE145370 revealed substantial TAM infiltration within the ESCC microenvironment (Figure S2A), with high expression of CD163 and CD206 indicating an M2‐polarized state (Figure S2B). The proportion of macrophages was significantly higher in ESCC tissues than in NATs and increased with pathological stage (Figure S2C). CIBERSORT analysis of bulk RNA‐seq data from patients with ESCC demonstrated that high M2‐TAM infiltration was associated with significantly poorer prognosis compared with low infiltration (Figure 2C, D).

**FIGURE 2:**
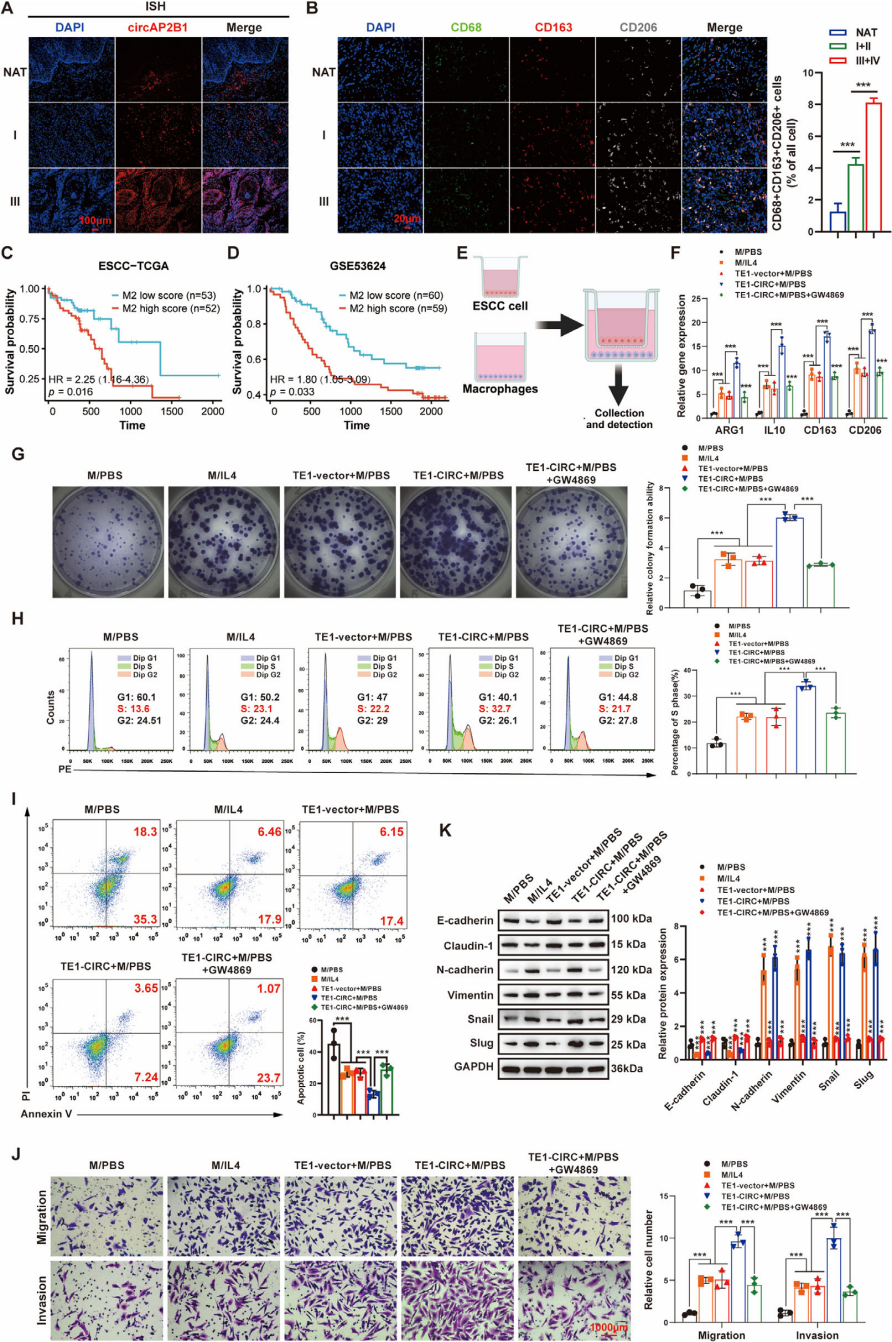
circAP2B1 Overexpression Correlates with M2‐TAM Infiltration and Promotes ESCC Progression. (A) Representative FISH images and quantification of circAP2B1 expression in ESCC tissues and NATs. Scale bar = 100 µm. (B) Representative multiplex immunofluorescence (MIF) images and quantification for CD68, CD163, and CD206 in ESCC tissues and NATs. Scale bar = 20 µm. (C, D) Kaplan‐Meier analysis of OS based on M2‐TAM infiltration abundance in ESCC patients from the TCGA‐ESCC (C) and GSE53624 (D) datasets. (E) Schematic diagram of the indirect co‐culture system between ESCC cells and macrophages. (F) RT‐qPCR analysis of M2 macrophage marker expression in macrophages co‐cultured with TE1‐vector or TE1‐circAP2B1 cells, or treated with PBS/IL‐4 controls. (G‐H) Colony formation assay (G) and Cell cycle analysis (H) by flow cytometry assessing the proliferation of TE1 cells co‐cultured with the indicated pre‐treated macrophages. (I) Apoptosis analysis by flow cytometry of TE1 cells co‐cultured with the indicated pre‐treated macrophages. (J) Transwell migration and invasion assays of TE1 cells co‐cultured with the indicated pre‐treated macrophages. Scale bar = 1000 µm. (K) Western blot analysis of EMT markers in TE1 cells co‐cultured with the indicated pre‐treated macrophages. ***p < 0.001.

To further delineate the immune landscape associated with circAP2B1 expression, single‐cell RNA sequencing was performed on three pairs of ESCC tissues exhibiting high versus low circAP2B1 expression. UMAP/t‐SNE clustering identified multiple immune cell subsets, including macrophages, T cells, natural killer (NK) cells, B cells, and dendritic cells (Figure S2D). The proportion of macrophages was significantly higher in circAP2B1‐high tumors than in circAP2B1‐low tumors, whereas no significant differences were observed in other immune subsets, including CD8^+^ T cells, NK cells, or B cells (Figure S2E, F). Moreover, gene set enrichment analysis for the M2 signature revealed a significantly elevated M2 score in macrophages from circAP2B1‐high tumors (Figure S2G), indicating that circAP2B1 specifically promotes M2 polarization of TAMs.

An indirect co‐culture system was employed to assess macrophage polarization and its effects on ESCC cell proliferation, apoptosis, migration, invasion, and EMT (Figure 2E). Compared with macrophages co‐cultured with vector‐control TE1 cells, those co‐cultured with circAP2B1‐overexpressing TE1 cells exhibited significantly increased expression of M2 markers (ARG1, IL10, CD163, CD206). This effect was abrogated by the exosome synthesis/release inhibitor GW4869 (Figure 2F). Macrophages treated with IL‐4 or phosphate‐buffered saline (PBS) served as positive and negative controls for M2 polarization, respectively. M2‐polarized macrophages significantly enhanced TE1 cell proliferation relative to the negative control. Similarly, macrophages co‐cultured with circAP2B1‐overexpressing TE1 cells promoted TE1 cell proliferation, an effect reversed by GW4869 (Figure 2G, H). M2‐polarized macrophages significantly inhibited TE1 cell apoptosis, as did macrophages co‐cultured with circAP2B1‐overexpressing cells; these effects were reversed by GW4869 (Figure 2I). Furthermore, M2‐polarized macrophages markedly promoted TE1 cell migration and invasion. Macrophages co‐cultured with circAP2B1‐overexpressing cells exhibited a similar pro‐metastatic effect, which was blocked by GW4869 (Figure 2J). At the molecular level, M2 macrophages upregulated mesenchymal markers (N‐cadherin, Vimentin, Snail, Slug) and downregulated epithelial markers (E‐cadherin, Claudin‐1) in TE1 cells. Co‐culture with circAP2B1‐overexpressing cells induced a comparable EMT phenotype, which was reversible by GW4869 (Figure 2K).

These results indicate that ESCC cell‐derived circAP2B1 may be transferred via exosomes to induce M2 macrophage activation, thereby promoting ESCC cell proliferation, migration, invasion, and EMT.

### 3.3 Tumor‐Derived Exosomal circAP2B1 Promotes ESCC Progression by Inducing M2 Polarization of TAMs

To determine whether ESCC cells constitute the primary source of circulating exosomal circAP2B1, exosomes were isolated from sorted epithelial cells (EpCAM^+^), total immune cells (CD45^+^), and stromal cells (CD45^–^EpCAM^–^) derived from ESCC tissues and paired NATs. RT‐qPCR analysis revealed that exosomes from ESCC epithelial cells contained significantly higher levels of circAP2B1 compared with exosomes from NAT epithelial cells, whereas no significant differences were observed in exosomes derived from immune or stromal cells between ESCC and NATs (Figure S3A). These results indicate that ESCC tumor epithelial cells are the major cellular source of exosomal circAP2B1.

Given the established role of exosomes in intercellular communication [18, 19], it was hypothesized that circAP2B1 from ESCC cells could be delivered to macrophages via exosomes to modulate TAM polarization. To test this, exosomes were isolated from the conditioned medium of KYSE30 and TE1 cells. TEM revealed typical cup‐shaped vesicles with diameters ranging from 50 to 150 nm, and NTA demonstrated comparable size distributions (Figure 3A). WB analysis confirmed the presence of exosomal markers (CD9, CD63, CD81, ALIX, HSP70, TSG101), with whole cell lysates serving as controls (Figure 3B). Uptake of exosomes by macrophages was assessed using PKH26‐labeled (red) ESCC‐derived exosomes, which were efficiently internalized (Figure 3C). Overexpression of circAP2B1 in KYSE30 cells significantly increased its abundance in exosomes without altering intracellular circAP2B1 levels (Figure S3B). To directly confirm exosomal transfer of circAP2B1 from ESCC cells to macrophages, KYSE30 cells were pretreated with the exosome synthesis inhibitor GW4869 prior to co‐culture with THP‐1 macrophages or exosome isolation. GW4869 treatment markedly reduced circAP2B1 levels in KYSE30‐derived exosomes and abolished the corresponding increase in circAP2B1 levels in co‐cultured THP‐1 macrophages (Figure S3C). Consistently, treatment of THP‐1 macrophages with purified Exo‐circAP2B1 derived from KYSE30 cells significantly elevated circAP2B1 levels in macrophages, whereas pre‐treatment of KYSE30 cells with GW4869 fully reversed this effect (Figure S3D).

**FIGURE 3:**
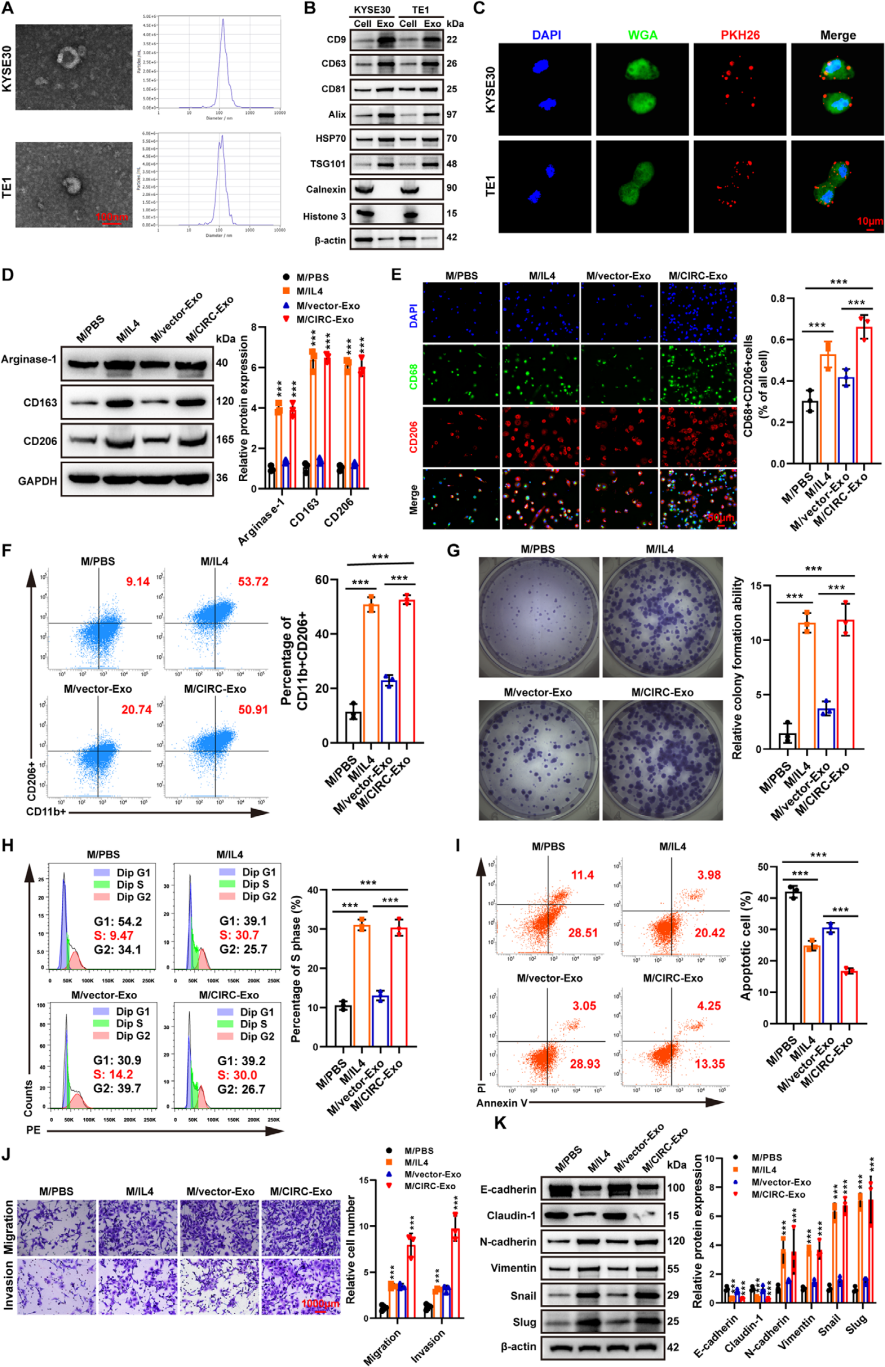
Tumor‐Derived Exosomal circAP2B1 Promotes ESCC Progression by Inducing M2 Polarization of TAMs. (A) Representative transmission electron microscopy (TEM) images and nanoparticle tracking analysis (NTA) of exosomes isolated from ESCC cell culture supernatants. (B) Western blot analysis of exosomal markers (CD63, ALIX, CD9, CD81, HSP70, TSG101) in isolated exosomes and whole cell lysates (WCL). Data are representative of three independent experiments. (C) Representative immunofluorescence (IF) images showing the internalization of PKH26‐labeled (red) ESCC‐derived exosomes by macrophages. Scale bar = 10 µm. (D‐F) Western blot (D), Immunofluorescence (E) (Scale bar = 50 µm), and Flow cytometry analysis (F) of M2 macrophage markers in macrophages treated with tumor‐derived Exo‐circAP2B1. (G, H) Colony formation assay (G) and Cell cycle analysis (H) assessing the proliferation of KYSE30 cells co‐cultured with macrophages pre‐treated with Exo‐circAP2B1. (I) Apoptosis analysis of KYSE30 cells co‐cultured with macrophages pre‐treated with Exo‐circAP2B1. (J, K) Transwell migration (Scale bar = 1000 µm) and invasion assays (J) and Western blot analysis (K) of EMT markers in KYSE30 cells co‐cultured with macrophages pre‐treated with Exo‐circAP2B1. ***p < 0.001.

The effect of tumor‐derived Exo‐circAP2B1 on macrophage polarization was then evaluated. Exo‐circAP2B1 significantly upregulated M2 markers, including ARG‐1, CD163, and CD206 (Figure 3D). Multiplex immunofluorescence analysis for CD68/CD206 (Figure 3E) and flow cytometry for CD11b/CD206 (Figure 3F) confirmed that Exo‐circAP2B1 treatment markedly increased the proportion of M2 macrophages. Functionally, macrophages exposed to Exo‐circAP2B1 significantly promoted KYSE30 cell proliferation (Figure 3G, H), inhibited apoptosis (Figure 3I), and enhanced migration and invasion (Figure 3J). These macrophages also induced an EMT phenotype in KYSE30 cells, characterized by upregulation of mesenchymal markers and downregulation of epithelial markers (Figure 3K). These in vitro findings demonstrate that ESCC cells transfer circAP2B1 to macrophages via exosomes to promote their M2 polarization, thereby facilitating tumor malignant progression.

### 3.4 Tumor‐Derived Exosomal circAP2B1 Promotes M2 Polarization by Enhancing Mitochondrial Homeostasis in TAMs

To investigate the mechanism by which tumor‐derived Exo‐circAP2B1 promotes M2 polarization, RNA sequencing was performed on macrophages treated with Exo‐circAP2B1. Volcano plot (Figure 4A) and heatmap (Figure 4B) analyses identified 376 upregulated and 520 downregulated genes in Exo‐circAP2B1‐treated macrophages, among which MFN2 was one of the most significantly upregulated. Gene Ontology analysis indicated that Exo‐circAP2B1 treatment significantly modulated biological processes associated with mitochondrial fusion and ATP generation (Figure 4C), implicating a role in mitochondrial regulation. To assess mitochondrial morphology, samples from three independent experiments were examined using high‐magnification TEM (50 000 ×). Exo‐circAP2B1‐treated macrophages displayed markedly elongated and fused mitochondria (Figure 4D). Quantitative analyses demonstrated that Exo‐circAP2B1 significantly increased the average mitochondrial area, the number of cristae per mitochondrion, and the mitochondrial aspect ratio compared to controls (Figure 4D). Functional assays further confirmed that Exo‐circAP2B1 treatment significantly elevated intracellular ATP levels and enhanced mitochondrial OCR, including basal respiration, maximal respiration, ATP production, and spare respiratory capacity; these effects were abrogated by GW4869 (Figure 4E, F). Moreover, Exo‐circAP2B1 from tumor cells markedly decreased ROS levels and increased mitochondrial membrane potential in macrophages, effects reversed by GW4869 (Figure 4G, I). In contrast, exosomes derived from circAP2B1‐knockdown cells (Exo‐sh‐circAP2B1) increased ROS levels and reduced mitochondrial membrane potential, with GW4869 further accentuating these changes (Figure 4H, J).

**FIGURE 4:**
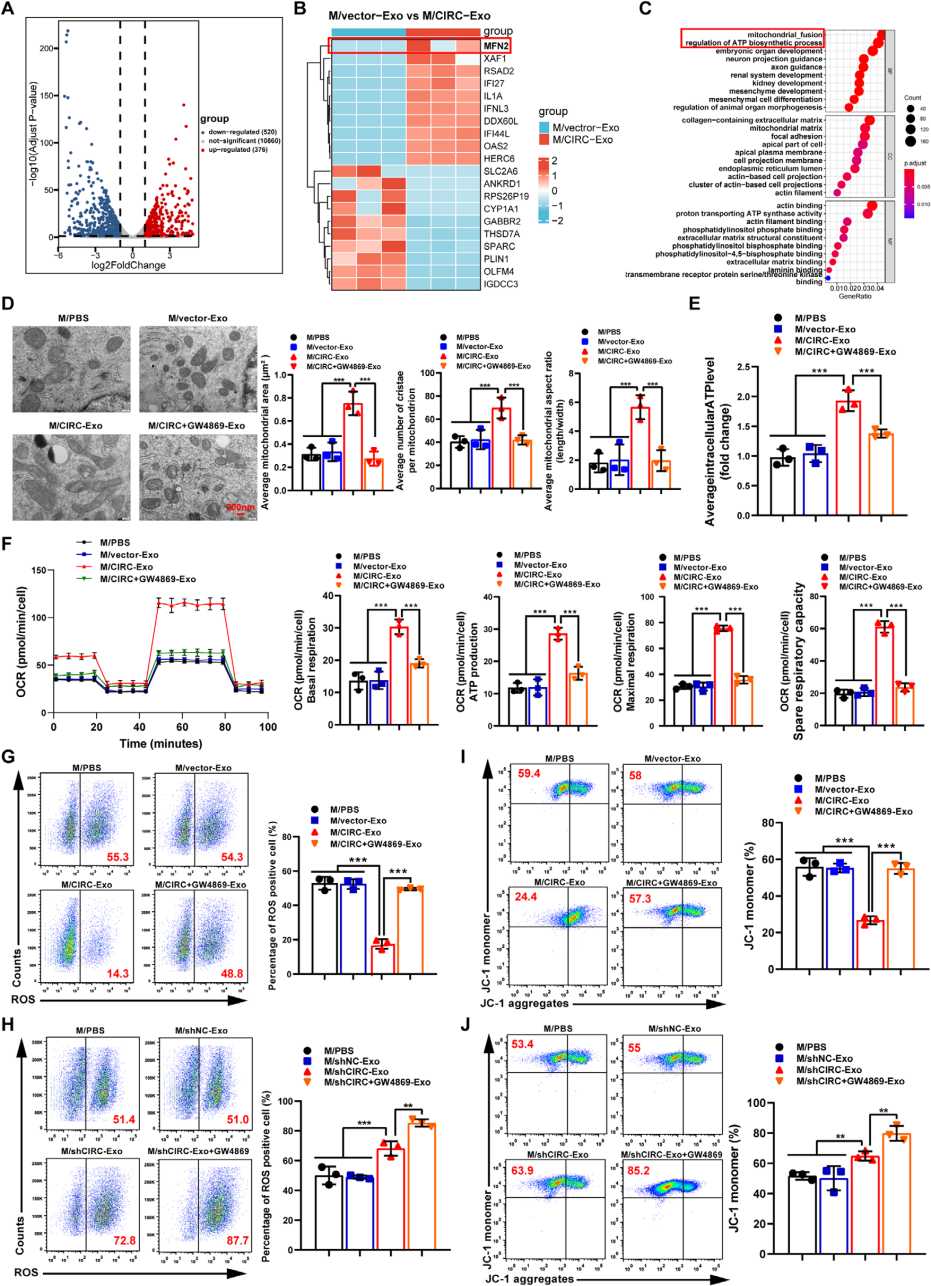
Tumor‐derived exosomal circAP2B1 promotes M2 polarization by enhancing mitochondrial homeostasis in TAMs. (A, B) Volcano plot (A) and Heatmap (B) of differentially expressed genes in macrophages after incubation with tumor‐derived Exo‐circAP2B1 (RNA‐seq). (C) Gene Ontology (GO) analysis of biological processes enriched for the differentially expressed genes. (D) Representative TEM images of mitochondria in macrophages treated with control exosomes or Exo‑circAP2B1 (50 000×; scale bar = 200 nm). Quantitative analysis of average mitochondrial area (µm^2^), cristae number per mitochondrion, and aspect ratio (length/width) is shown. Data are from three independent experiments (≥30 cells/condition, ≥10 mitochondria/cell; total ≥900 mitochondria per condition). (E) Intracellular ATP levels in macrophages treated as indicated. (F) Mitochondrial oxygen consumption rate (OCR) in macrophages, measuring basal respiration, maximal respiration, ATP production, and spare respiratory capacity. (G, I) Intracellular ROS levels (G) and mitochondrial membrane potential (I) in macrophages following treatment with Exo‑circAP2B1. (H, J) Intracellular ROS levels (H) and mitochondrial membrane potential (J) in macrophages following treatment with Exo‑sh‑circAP2B1. ***p < 0.001.

To establish a causal relationship between enhanced mitochondrial homeostasis and M2 polarization, rescue experiments were conducted using the mitochondrial ATP synthase inhibitor oligomycin. Exo‐circAP2B1 significantly upregulated M2 markers, including Arginase‐1, CD163, and CD206, at the protein level (Figure S4A), increased the proportion of CD68^+^CD206^+^ double‐positive cells (Figure S4B), and elevated the percentage of CD11b^+^CD206^+^ M2 macrophages as determined by flow cytometry (Figure S4C). Importantly, oligomycin treatment completely reversed all Exo‐circAP2B1‐induced increases in M2 markers, whereas GW4869 similarly blocked these effects. These results demonstrate that tumor‐derived Exo‐circAP2B1 enhances mitochondrial homeostasis in TAMs, which in turn drives M2 polarization.

### 3.5 The circAP2B1/ESRRA/KPNA1 Ternary Complex Translocates to the Nucleus to Promote MFN2 Transcription

To investigate the mechanism by which Exo‐circAP2B1 regulates mitochondrial homeostasis in macrophages, MFN2 expression was examined. RT‐qPCR (Figure 5A) and WB analysis (Figure 5B) confirmed that Exo‐circAP2B1 significantly upregulated MFN2 in macrophages, corroborating the RNA‐seq findings. It is hypothesized that Exo‐circAP2B1 enhances mitochondrial homeostasis by modulating MFN2 transcription. A pull‐down assay using a biotinylated circAP2B1 probe followed by LC‐MS/MS identified 125 proteins potentially bound specifically to circAP2B1, among which the transcription factor ESRRA exhibited the highest abundance (Figure 5C, D). Subsequent pull‐down (Figure 5E) and RIP assays (Figure 5F, Figure S5A) validated the specific interaction between circAP2B1 and ESRRA in macrophages. RNAfold prediction suggested four potential structural domains in circAP2B1 (Figure 5G), and domain mutation experiments identified the HR2 domain as essential for the circAP2B1–ESRRA interaction (Figure 5H, Figure S5B). Truncation mutagenesis further revealed that the 1–200 amino acid region of ESRRA mediates its binding to circAP2B1 (Figure 5I, J).

**FIGURE 5:**
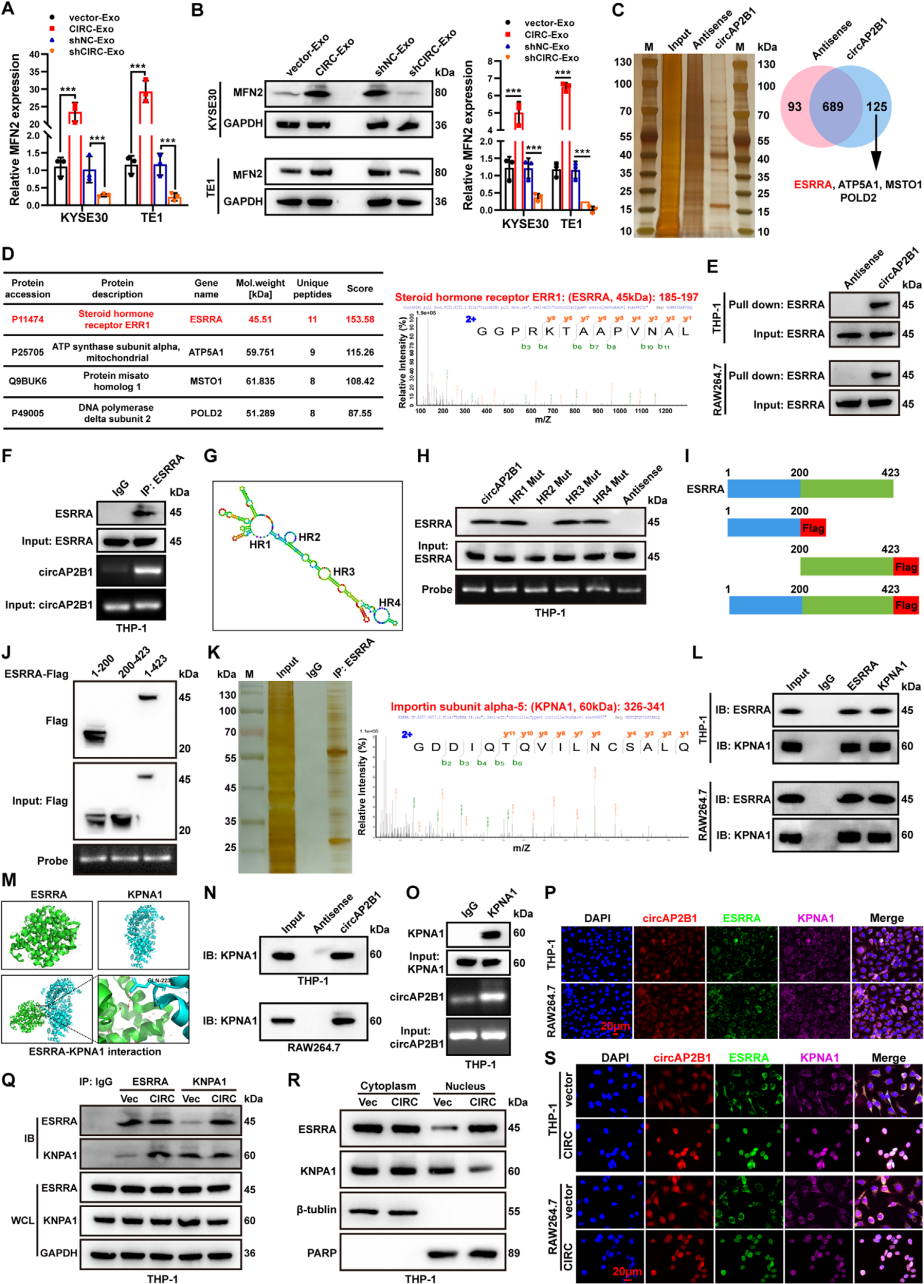
The circAP2B1/ESRRA/KPNA1 Ternary Complex Translocates to the Nucleus to Promote MFN2 Transcription. (A, B) RT‐qPCR (A) and Western blot (B) analysis of MFN2 expression in macrophages treated with tumor‐derived Exo‐circAP2B1. Data are representative of three independent experiments. (C) Silver staining and LC‐MS/MS analysis of proteins pulled down by biotinylated circAP2B1 probe. (D) Left: Details of the top 5 proteins specifically binding to circAP2B1. Right: Secondary mass spectrum of the ESRRA peptide. (E, F) Pull‐down (E) and RIP (F) assays validating the interaction between circAP2B1 and ESRRA. Data are representative of three independent experiments. (G) Predicted secondary structure of circAP2B1 from the RNAfold Web Server. (H) Pull‐down assay identifying the HR2 domain of circAP2B1 responsible for binding ESRRA using domain mutants. Data are representative of three independent experiments. (I) Schematic of Flag‐tagged ESRRA truncation plasmids. (J) Pull‐down assay identifying the N‐terminal (1‐200 aa) region of ESRRA responsible for binding circAP2B1. Data are representative of three independent experiments. (K) Left: Silver staining and LC‐MS/MS of proteins co‐immunoprecipitated with ESRRA. Right: Secondary mass spectrum of the KPNA1 peptide. (L) Co‐IP assay validating the interaction between ESRRA and KPNA1. Data are representative of three independent experiments. (M) Predicted molecular interaction model between ESRRA and KPNA1 from PyMol. (N, O) Pull‐down (N) and RIP (O) assays validating the interaction between circAP2B1 and KPNA1. Data are representative of three independent experiments. (P) FISH‐IF showing the co‐localization of circAP2B1, ESRRA, and KPNA1. Scale bar = 20 µm. (Q) Co‐IP assay showing that circAP2B1 enhances the ESRRA‐KPNA1 interaction. Data are representative of three independent experiments. (R, S) Western blot (R) of nuclear/cytoplasmic fractions and FISH‐IF (S) analysis showing that circAP2B1 promotes nuclear translocation of ESRRA and KPNA1. Scale bar = 20 µm. Data are representative of three independent experiments.

Given that karyopherins regulate the nuclear import of transcription factors [20, 21], ESRRA's interactome was explored. LC‐MS/MS analysis of ESRRA immunoprecipitates identified karyopherin subunit alpha 1 (KPNA1) as a top interactor (Figure 5K). Co‐IP confirmed the ESRRA–KPNA1 interaction in THP‐1 and RAW264.7 macrophages (Figure 5L), and PyMol modeling predicted the interface of interaction (Figure 5M). Pull‐down (Figure 5N) and RIP assays (Figure 5O, Figure S5C) demonstrated that circAP2B1 also interacts with KPNA1, suggesting the formation of a circAP2B1/ESRRA/KPNA1 ternary complex.

To examine the subcellular localization of circAP2B1 in recipient macrophages, RNA FISH was performed in THP‐1 and RAW264.7 cells. As shown in Figure S5D, circAP2B1 was predominantly nuclear, indicating that following exosomal transfer, circAP2B1 translocates to the nucleus, where it can participate directly in chromatin interactions and transcriptional regulation. Consistent with its nuclear localization, FISH combined with immunofluorescence revealed co‐localization of circAP2B1, ESRRA, and KPNA1 in the cytoplasm (Figure 5P). Co‐IP further demonstrated that circAP2B1 enhances the ESRRA–KPNA1 interaction (Figure 5Q, Figure S5E). Nuclear–cytoplasmic fractionation (Figure 5R, Figure S5F) and FISH–immunofluorescence (Figure 5S) confirmed that circAP2B1 facilitates the nuclear translocation of both ESRRA and KPNA1.

SPR was employed to quantify the binding affinity of circAP2B1 for the preformed ESRRA/KPNA1 complex. Concentration‐dependent binding responses were observed over the range of 7–224 µM (Figure S5G,H). Langmuir fitting yielded a dissociation constant (*K*D) of 26.68 µM, *R_max_* of 22.84 RU, and *R^2^* of 0.9987. Control injections with ESRRA or KPNA1 alone produced markedly lower responses, confirming specific binding of circAP2B1 to the ternary complex (Figure S5I).

Finally, clinical relevance was assessed by multiplex immunofluorescence on ESCC tumor sections (n  =  79 per group). circAP2B1‐high tumors exhibited significantly higher ESRRA and MFN2 fluorescence intensities in CD206^+^ TAMs compared to circAP2B1‐low tumors (Figure S5J), indicating that elevated circAP2B1 expression correlates with increased ESRRA and MFN2 protein levels in TAMs within the TME. These results demonstrate that the circAP2B1/ESRRA/KPNA1 ternary complex translocates to the nucleus to promote MFN2 transcription, as supported by cellular localization studies, Co‐IP, biophysical SPR measurements, and clinical tissue analyses.

### 3.6 circAP2B1 Promotes MFN2 Transcription

Overexpression of circAP2B1 in macrophages led to a marked upregulation of MFN2 at both the mRNA and protein levels, whereas knockdown of ESRRA or KPNA1 effectively abrogated this effect (Figure 6A, B). Conversely, circAP2B1 knockdown reduced MFN2 expression, which was fully rescued by overexpression of ESRRA or KPNA1 (Figure 6C, D). RNA pull‐down assays confirmed the specificity of the circAP2B1 probe (Figure 6E) and demonstrated a direct interaction between circAP2B1 and the MFN2 promoter (Figure 6F, G). ChIRP further revealed significant enrichment of circAP2B1 at the ‐600 to ‐400 region of the MFN2 promoter (Figure 6H), a finding corroborated by pull‐down assays showing specific binding to this promoter fragment (Figure 6I) and validated by luciferase reporter assays (Figure 6J). Truncation mutagenesis of circAP2B1 identified the 241–320 nt region as essential for its promoter interaction(Figure 6K, L), which was confirmed by subsequent luciferase assays (Figure 6M).

**FIGURE 6:**
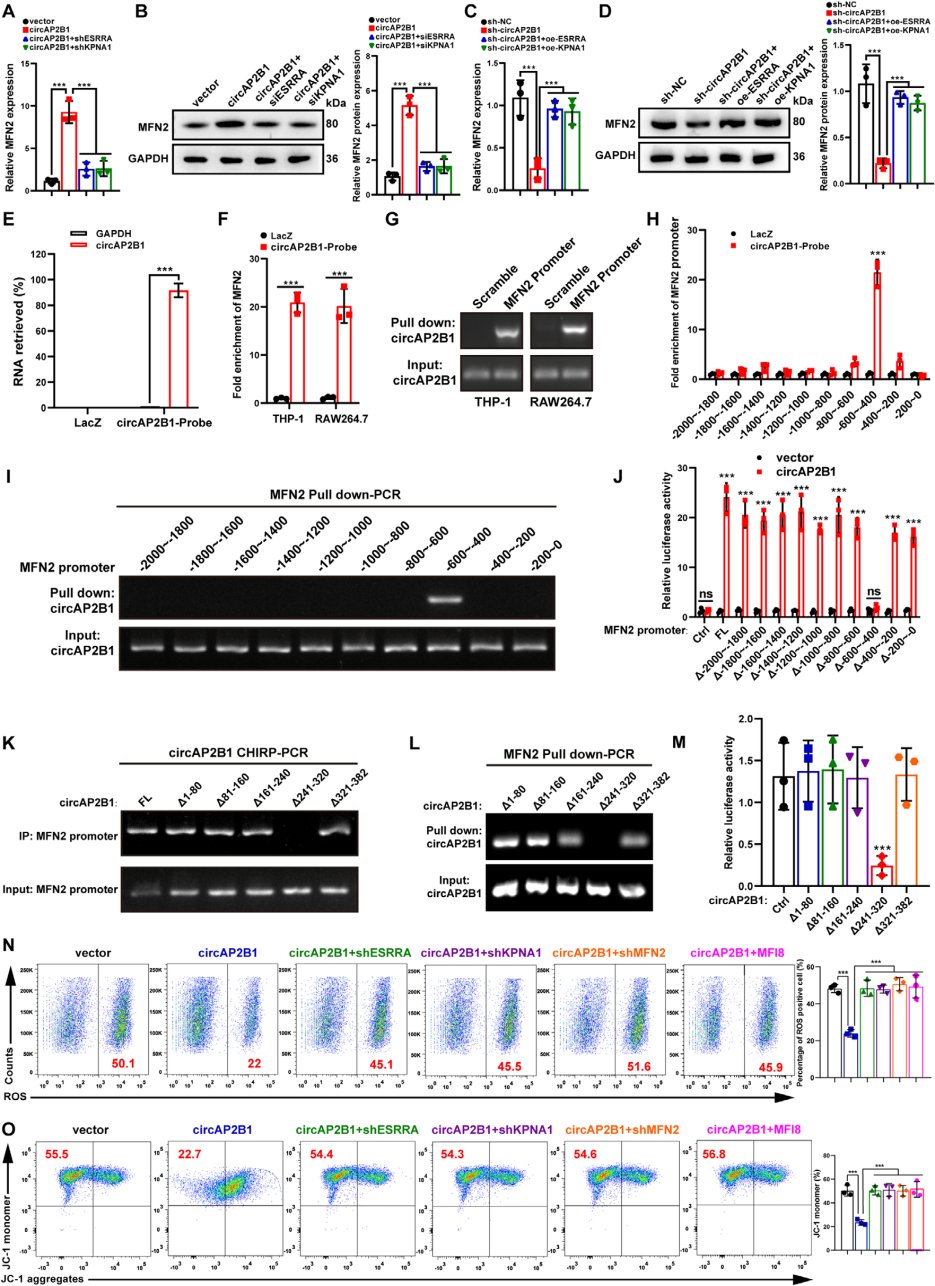
circAP2B1 Promotes MFN2 Transcription. (A, B) RT‐qPCR (A) and Western blot (B) analysis of MFN2 expression in macrophages under the indicated conditions (circAP2B1 OE with/without ESRRA or KPNA1 KD). (C, D) RT‐qPCR (C) and Western blot (D) analysis of MFN2 expression in macrophages under the indicated conditions (circAP2B1 KD with/without ESRRA or KPNA1 OE). (E) RNA pull‐down assay verifying the specificity of the circAP2B1 probe. (F, G) CHIRP‐qPCR (F) and Pull‐down‐PCR (G) assays detecting the interaction between circAP2B1 and the MFN2 promoter. Data are representative of three independent experiments. (H, I) CHIRP‐qPCR (H) and Pull‐down‐PCR (I) assays mapping the circAP2B1 binding region on the MFN2 promoter. Data are representative of three independent experiments. (J) Dual‐luciferase reporter assay for the MFN2 promoter region interacting with circAP2B1. (K, L) CHIRP‐PCR (K) and Pull‐down‐PCR (L) assays mapping the MFN2‐binding region within the circAP2B1 sequence. Data are representative of three independent experiments. (M) Dual‐luciferase reporter assay for the circAP2B1 sequence region interacting with the MFN2 promoter. (N, O) ROS levels (N) and Mitochondrial membrane potential (O) in macrophages under the indicated conditions. ***p < 0.001.

To establish the functional relevance of the circAP2B1/ESRRA/KPNA1/MFN2 axis in mitochondrial homeostasis, rescue experiments were conducted. circAP2B1 overexpression significantly decreased intracellular ROS levels and increased mitochondrial membrane potential; these effects were completely reversed by knockdown of ESRRA, KPNA1, or MFN2, as well as by treatment with the MFN2 inhibitor MFI‐8 (Figure 6N, O). Similarly, circAP2B1 overexpression elevated cellular ATP levels and promoted mitochondrial fusion, whereas these phenotypes were abolished by depletion of ESRRA, KPNA1, or MFN2, or by MFI‐8 treatment (Figure S6A, B). These results demonstrate that the circAP2B1/ESRRA/KPNA1/MFN2 axis is both necessary and sufficient for circAP2B1‐mediated enhancement of mitochondrial function, indicating that circAP2B1 directly engages the MFN2 promoter to drive its transcription and thereby maintain mitochondrial homeostasis.

### 3.7 circAP2B1 Recruits ESRRA to the MFN2 Promoter to Initiate Transcription

Given these findings, this study further investigated whether ESRRA directly regulates MFN2 transcription by binding to its promoter. Pull‐down assays using either an anti‐ESRRA antibody or an MFN2 promoter probe confirmed a direct ESRRA–MFN2 promoter interaction (Figure 7A, B). Overexpression of circAP2B1 enhanced, while its knockdown impaired, the recruitment of ESRRA to the MFN2 promoter (Figure 7C, D). Bioinformatic analysis using the JASPAR database identified a potential ESRRA binding motif and three putative binding sites within the MFN2 promoter (Figure 7E, F). ChIP‐PCR analysis demonstrated that ESRRA specifically occupies Site 2 of the promoter (Figure 7G, H), which was validated functionally by luciferase reporter assays using wild‐type and mutant Site 2 constructs (Figure 7I–K). Truncation mutagenesis further revealed that the 1–200 amino acid region of ESRRA mediates its promoter binding (Figure 7L). These results establish that circAP2B1 facilitates the recruitment of ESRRA to a specific site on the MFN2 promoter, thereby positively regulating MFN2 transcription.

**FIGURE 7:**
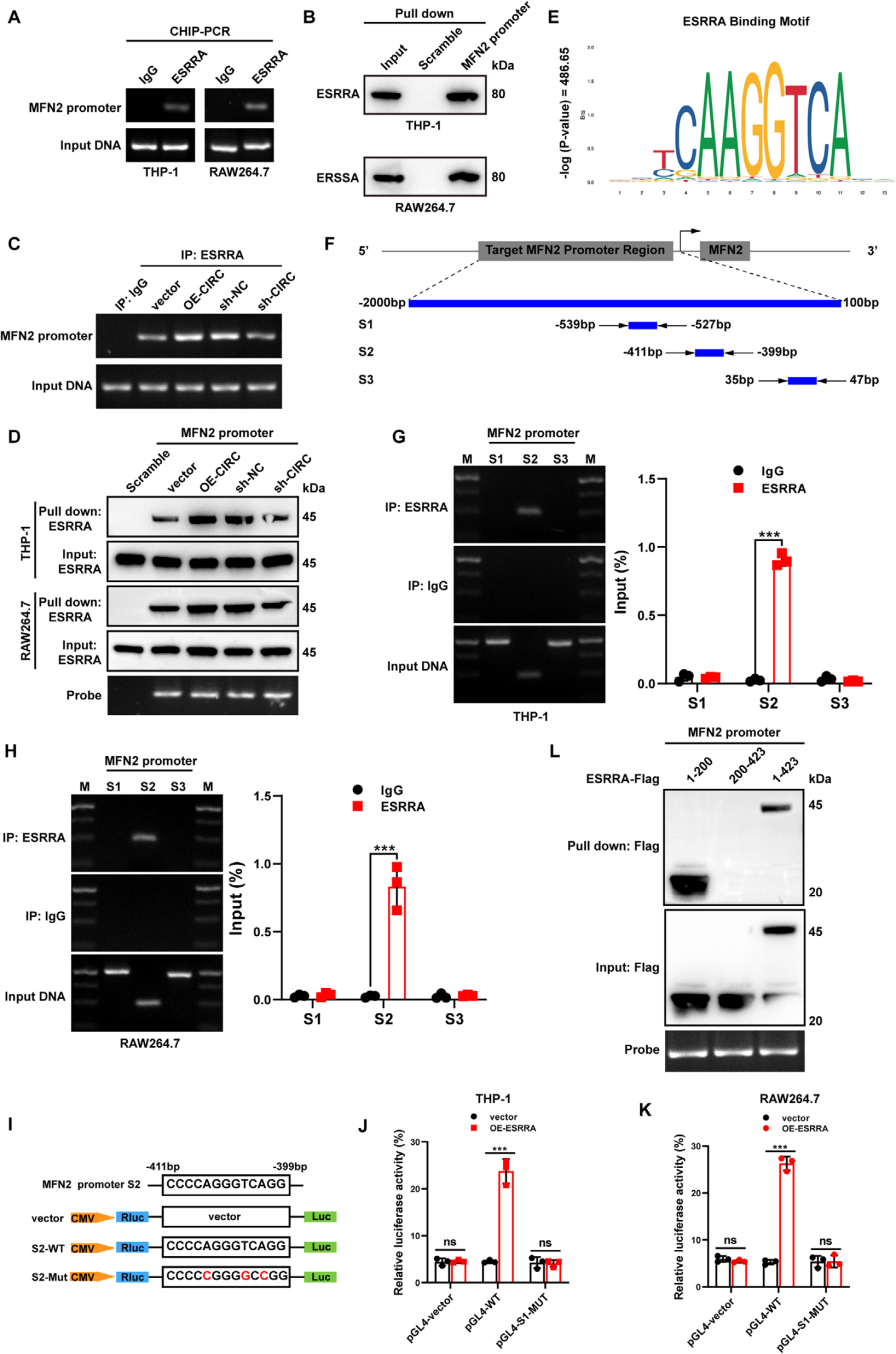
circAP2B1 Recruits ESRRA to the MFN2 Promoter to Initiate Transcription. (A‐B) ChIP‐PCR (A) and Pull‐down (B) assays detecting the interaction between ESRRA and the MFN2 promoter. Data are representative of three independent experiments. (C, D) ChIP‐PCR (C) and Pull‐down (D) assays evaluating the effect of circAP2B1 OE or KD on the ESRRA‐MFN2 promoter binding. Data are representative of three independent experiments. (E) ESRRA binding motif obtained from the JASPAR database. (F) Schematic of three potential ESRRA binding sites in the MFN2 promoter region. (G, H) ChIP‐PCR analysis identifying the specific ESRRA binding site (Site 2) on the MFN2 promoter. Data are representative of three independent experiments. (I) Schematic of wild‐type and mutant sequences of Site 2 used in luciferase reporters. (J, K) Dual‐luciferase reporter assays validating Site 2 as the functional ESRRA binding site. (L) Pull‐down assay identifying the N‐terminal (1‐200 aa) region of ESRRA responsible for binding the MFN2 promoter. Data are representative of three independent experiments. ***p < 0.001 and ns, not significant.

### 3.8 Tumor‐Derived Exosomal circAP2B1 Promotes ESCC Progression In Vivo by Inducing M2‐TAM Polarization

To validate the functional role of the circAP2B1/ESRRA/KPNA1/MFN2 axis in vivo, subcutaneous xenograft and lung metastasis mouse models were employed. KYSE30 cells overexpressing circAP2B1 or vector control were co‐cultured with THP‐1 macrophages transfected with shNC, shKPNA1, or shMFN2. The pre‐conditioned macrophages were then mixed with KYSE30 cells and injected either subcutaneously or intravenously to establish the respective tumor models.

In the subcutaneous xenograft model, macrophages pre‐treated with Exo‐circAP2B1 from KYSE30 cells significantly accelerated tumor growth, as evidenced by increased tumor growth rate (Figure 8A), tumor weight (Figure 8B), and tumor volume (Figure 8C) compared with controls. These pro‐tumorigenic effects were abrogated upon inhibition of exosome secretion by GW4869 or by knockdown of KPNA1 or MFN2 in the macrophages. Multiplex immunofluorescence of the TME revealed that Exo‐circAP2B1‐conditioned macrophages markedly enhanced M2‐TAM infiltration (CD163^+^, CD206^+^), which was reversed by GW4869 or KPNA1/MFN2 depletion (Figure 8D). Immunohistochemical analysis further demonstrated elevated MFN2 expression, increased KI‐67 proliferation marker levels, and induction of EMT in the xenografts conditioned with Exo‐circAP2B1, whereas these effects were abolished by the respective interventions (Figure 8E).

**FIGURE 8:**
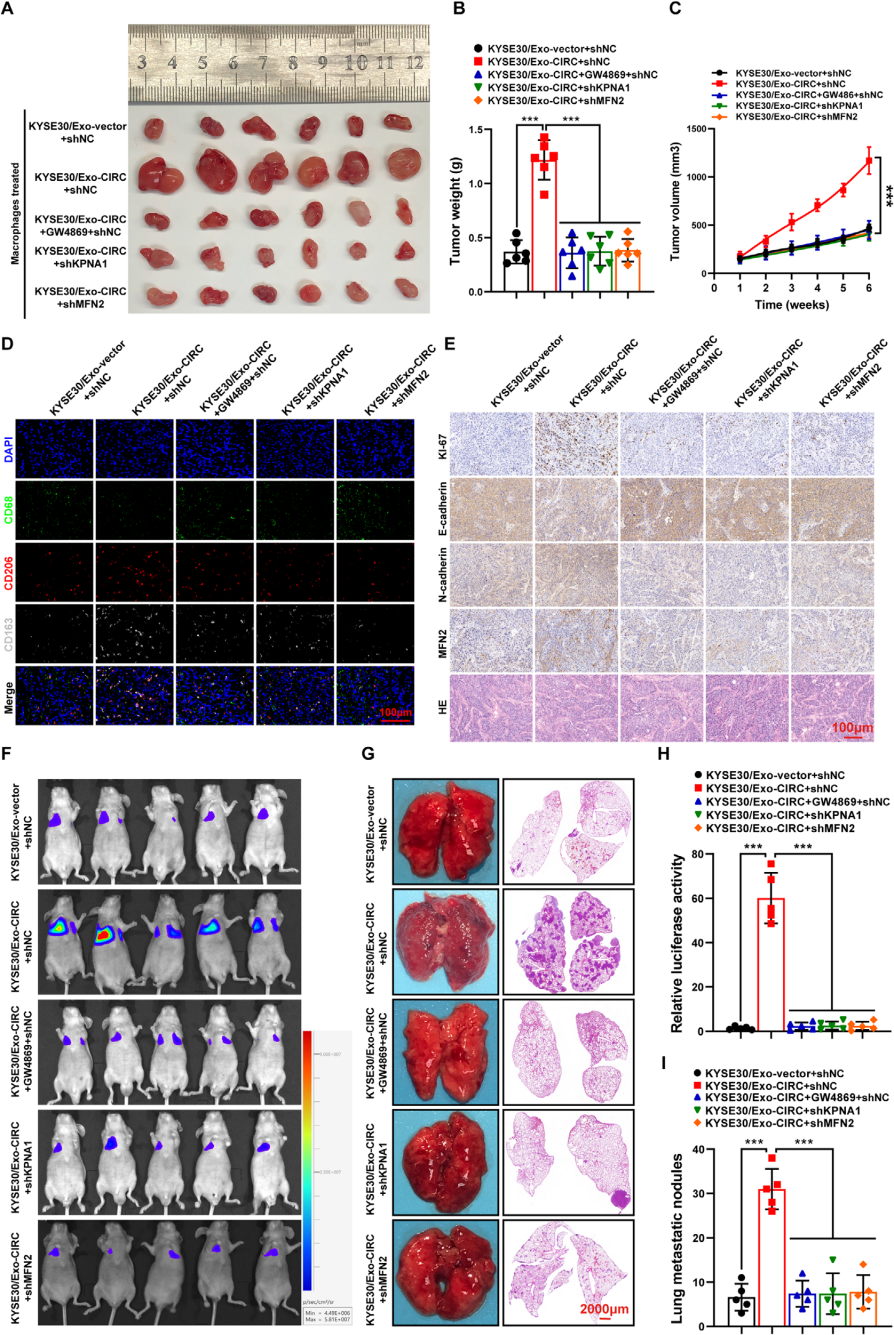
Tumor‐Derived Exosomal circAP2B1 Promotes ESCC Progression In Vivo by Inducing M2‐TAM Polarization. (A) Photographs of subcutaneous xenograft tumors from each group (n = 6). (B) Tumor weight and (C) Tumor volume of subcutaneous xenografts (n = 6). (D) Representative MIF images (CD68/CD163/CD206) of subcutaneous tumor sections. (E) Representative IHC images for Ki67, E‐cadherin, N‐cadherin, and MFN2 in subcutaneous tumor sections. (F) In vivo bioluminescence imaging of lungs from the metastatic model (n = 5). (G) H&E staining of lung tissues from the metastatic model. (H) Quantification of lung fluorescence intensity and (I) Number of lung metastatic nodules (n = 5). ***p < 0.001.

To assess the impact of the circAP2B1/ESRRA/KPNA1/MFN2 axis on mitochondrial homeostasis within TAMs in vivo, TAMs were isolated from subcutaneous xenografts for functional analysis. TEM revealed that, compared with controls, TAMs from the Exo‐circAP2B1‐treated group exhibited significantly increased mitochondrial area, cristae density, and aspect ratio, indicative of enhanced mitochondrial fusion (Figure S7A). Correspondingly, these TAMs displayed elevated ATP levels (Figure S7B), decreased ROS accumulation (Figure S7C), and increased mitochondrial membrane potential (JC‐1 aggregates) (Figure S7D). Notably, knockdown of KPNA1 or MFN2 completely reversed these mitochondrial phenotypes, restoring morphology and function to baseline levels, thereby providing direct in vivo evidence that the circAP2B1/ESRRA/KPNA1/MFN2 axis modulates mitochondrial homeostasis in TAMs.

In the lung metastasis model, pretreatment of macrophages with Exo‐circAP2B1 significantly increased lung tumor burden, as demonstrated by higher lung fluorescence intensity (Figure 8F) and greater numbers of metastatic nodules (Figure 8G–I). These prometastatic effects were reversed by GW4869 treatment or KPNA1/MFN2 knockdown. Multiplex immunofluorescence (Figure S7E) and IHC analysis (Figure S7F) of lung metastases confirmed enhanced M2‐TAM infiltration, upregulation of MFN2 and KI‐67, and EMT induction in response to Exo‐circAP2B1, all of which were inhibited by the respective interventions.

These in vivo findings corroborate our in vitro data, demonstrating that ESCC‐derived exosomal circAP2B1 promotes tumor growth and metastasis by enhancing mitochondrial homeostasis to drive M2 polarization of TAMs.

## 4 Discussion

This study systematically delineates the molecular mechanism through which ESCC‐derived exosomal circAP2B1 induces M2 polarization of macrophages via mitochondrial metabolic reprogramming, thereby facilitating ESCC progression. The principal findings are as follows: (1) circAP2B1 is markedly upregulated in both tumor tissues and serum of patients with ESCC and exhibits a strong association with poor prognosis; (2) ESCC cells transmit circAP2B1 to TAMs through exosomes, promoting the assembly of a circAP2B1/ESRRA/KPNA1 ternary complex that drives nuclear translocation of the transcription factor ESRRA; (3) Nuclear ESRRA binds to the MFN2 promoter to activate its transcription, thereby enhancing mitochondrial fusion and ATP production, which in turn facilitates M2 polarization of TAMs; and (4) Targeted inhibition of circAP2B1 or its downstream effectors (ESRRA, KPNA1, or MFN2) substantially suppresses the M2 phenotype of TAMs and impedes ESCC malignant progression. These findings not only advance the understanding of regulatory mechanisms within the ESCC immune microenvironment but also offer novel insights for the development of intervention strategies targeting the metabolism‐immune axis.

In recent years, the regulatory functions of exosomal circRNAs within the TME have attracted considerable attention. Accumulating evidence indicates that tumor‐derived exosomal circRNAs can modulate immune cell function. For example, circUHRF1 suppresses NK cell activity to promote hepatocellular carcinoma progression [22], whereas circEML4 enhances lung cancer metastasis by inducing M2 macrophage polarization [23]. The present study reveals a distinctive mechanism in which circAP2B1, through exosomal delivery, reprograms mitochondrial metabolism in TAMs, thereby broadening the functional repertoire of circRNAs in tumor immune evasion. Importantly, the regulation of mitochondrial homeostasis by circAP2B1 is dependent on its interaction with ESRRA and KPNA1. This observation suggests that circRNAs may serve as “molecular scaffolds,” coordinating transcription factors and nuclear transport proteins to spatiotemporally regulate target gene expression. This mechanism bears conceptual resemblance to a recent report demonstrating that circRILPL1 binds to the nuclear import receptor IPO7 to facilitate YAP nuclear translocation [24]. Nevertheless, the present study reveals a previously unrecognized, central role for a circRNA in modulating mitochondrial metabolism within TAMs.

Although tumor cells frequently preferentially utilize glycolysis for energy production even under normoxic conditions (the Warburg effect), oxidative phosphorylation (OXPHOS) is not universally compromised and may be enhanced in certain cancers [25, 26]. For instance, Zhan et al. demonstrated that circPUM1 promotes ESCC progression by stabilizing mitochondrial complex III, thereby augmenting ATP production through OXPHOS [27]. Metabolic programs of both tumor cells and TAMs undergo reprogramming, often in a coordinated manner that shapes their functional phenotypes and orchestrates the complex process of tumor progression [28, 29]. Macrophage metabolic signatures are closely aligned with their functional states: M1 activation is typically associated with a shift from OXPHOS to aerobic glycolysis, whereas M2 polarization relies more heavily on an intact tricarboxylic acid (TCA) cycle and functional OXPHOS to sustain energy production [30, 31]. Accordingly, mitochondrial homeostasis is intimately linked to macrophage polarization. MFN2, a key mediator of mitochondrial fusion, enhances OXPHOS efficiency and mitigates ROS accumulation, thereby facilitating the M2 phenotype [32, 33]. The present data confirm that circAP2B1‐mediated transcriptional activation of MFN2 significantly improves mitochondrial function in macrophages, fully consistent with the metabolic requirements of M2 macrophages. Moreover, ESRRA, a member of the nuclear receptor family, has been implicated in the reprogramming of macrophage lipid metabolism [34, 35]; however, its role in circRNA‐mediated regulation of mitochondrial function had remained unclear. This study identifies ESRRA as a direct transcriptional activator of MFN2 and delineates the mechanism by which circAP2B1 controls ESRRA nuclear translocation, thereby providing new evidence for the interplay between metabolic and epigenetic regulatory networks.

This study further highlights the dual clinical relevance of circAP2B1 in ESCC: its characteristically elevated serum levels represent a novel non‐invasive diagnostic biomarker, whereas its regulation of TAM M2 polarization and mitochondrial function to remodel the TME offers new potential targets for immunotherapeutic intervention. Although limitations exist regarding the complexity of animal models and the comprehensive understanding of mitochondrial regulatory networks, future efforts—including the development of novel therapeutics targeting this pathway and further elucidation of the interactions between mitochondrial metabolism and immune cell function—are anticipated to advance combined therapeutic strategies based on metabolic‐immune regulation, thereby establishing a critical theoretical foundation for precision immunotherapy in ESCC.

Several limitations warrant consideration. First, the co‐injection model employed in the in vivo experiments, while effective, represents an artificial initiation that does not fully recapitulate the dynamic recruitment of macrophages during natural tumor development [36]. Second, all animal experiments were conducted in immune‐deficient BALB/c nude mice, which lack functional T cells; consequently, the broader immunomodulatory effects of circAP2B1 within a fully competent immune system remain to be elucidated [37]. Third, the clinical sample cohort was derived from a single institution, highlighting the need for multicenter prospective studies to validate the diagnostic and prognostic utility of serum exosomal circAP2B1 [38]. Fourth, owing to sample limitations and temporal constraints, higher‐resolution colocalization images (e.g., orthogonal views) could not be obtained, nor could in vivo loss‐of‐function experiments (e.g., macrophage‐specific knockout of ESRRA, KPNA1, or MFN2) be performed. Future investigations employing immunocompetent mouse models and advanced imaging methodologies will be essential to confirm the physiological relevance of the circAP2B1/ESRRA/KPNA1/MFN2 axis [39].

## 5 Conclusions

This study elucidates a previously unrecognized mechanism by which ESCC‐derived exosomal circAP2B1 drives tumor progression through the promotion of TAM M2 polarization. Acting as a molecular scaffold, circAP2B1 facilitates nuclear translocation of the ESRRA/KPNA1 complex, activates MFN2 transcription, and enhances mitochondrial homeostasis, thereby fostering an immunosuppressive microenvironment. These findings not only introduce a novel serum biomarker for ESCC diagnosis but also establish a conceptual foundation for the development of therapeutic strategies targeting the tumor metabolic‐immune axis.

## Author Contributions

L.K. and F.G. conceived and designed the experiments, secured funding for the project, and supervised the research. W.Y.R. and W.Y.Y. conducted the majority of the experiments. L.K. and W.Y.R. drafted the manuscript and prepared all figures. L.R.H. collected the clinical tissues. L.R.H. and W.Y.Y. analyzed the data. L.R.H. and P.H.Y. assisted with the research and critically reviewed the paper. All authors have read and approved the final version of the paper.

## Funding

This work was supported by National Natural Science Foundation of China (No. 82503345), Sichuan Science and Technology Program (No. 2025ZNSFSC1898), and Research Fund of Sichuan Academy of Medical Sciences and Sichuan Provincial People's Hospital (No. 24QNPY012, No. 25QNPY007).

## Conflicts of Interest

The authors declare no conflicts of interest.

## Supporting information




**Supporting File**: advs76903‐sup‐0001‐SuppMat.docx.

## Data Availability

The data that support the findings of this study are openly available in Gene Expression Omnibus at https://www.ncbi.nlm.nih.gov/geo/, reference number GSE296251.
