# Supplementary material for: Tumor‐Derived Exosomal circAP2B1 Induces M2 Macrophage Polarization by Enhancing Mitochondrial Homeostasis to Promote Esophageal Squamous Cell Carcinoma Progression
Source: Adv Sci (Weinh). 2026 Jul 31;13(59):e76903. doi: 10.1002/advs.76903 (PMC13427234; doi:10.1002/advs.76903)
Supplement: Supplementary file 1 [file ADVS-13-e76903-s001.docx]

**Tumor-derived exosomal circAP2B1 induces M2 macrophage polarization by enhancing mitochondrial homeostasis to promote esophageal squamous cell carcinoma progression**

**Authors: Yiru Wang^1^, Gang Feng^2^, Youyu Wang^2^, Ruihao Liang^3^, Haixia Pan^1^, Kai Lei^2, *^**

**Affiliations:**

**^1^**Department of Oncology & Cancer Institute, Sichuan Academy of Medical Sciences, Sichuan Provincial People's Hospital, University of Electronic Science and Technology of China, Chengdu, 610072, China.

**^2^**Department of Thoracic Surgery, Sichuan Academy of Medical Sciences, Sichuan Provincial People's Hospital, University of Electronic Science and Technology of China, Chengdu, 610072, China.

**^3^**Department of Thoracic Surgery, Sun Yat-sen Memorial Hospital, Sun Yat-sen University, Guangzhou, 510120, China

**Running Title:** Exosomal circAP2B1 drives M2 polarization

**^*^Corresponding author:**

Prof. Kai Lei, Department of Thoracic Surgery, Sichuan Academy of Medical Sciences, Sichuan Provincial People's Hospital, University of Electronic Science and Technology of China, Chengdu, 610072, China. E-mail: leikai@scsrmyy5.wecom.work

**Supplementary Tables**

| **Table S1. The sequence of shRNAs** | |
| --- | --- |
| **Name** | **Sequence (5'-3')** |
| circAP2B1-shRNA1-Sense | GGGACAGUCCUGACUGCUACA |
| circAP2B1-shRNA1-antiSense | UGUACGUCGACCUGACAGGGU |
| MFN2-shRNA3-Sense | GGACAUCAUCUACAACGAGAA |
| MFN2-shRNA3-antiSense | UUCUGCAUGAGGAACUACAGC |
| KPNA1-shRNA-Sense | GCAUGAAGACCUUCGACAACU |
| KPNA1-shRNA- antiSense | AGUUAGUCGAAGAUCCUUGAC |
| ESRRA-shRNA-Sense | GGACAUCAAGGAGUACGUCAU |
| ESRRA-shRNA- antiSense | AUGCAUGAGGAACUACAGGU |

| **Table S2. Primer sequences for RT-qPCR** | | |
| --- | --- | --- |
| **Gene name** | **Primer** | Sequence (5'-3') |
| GAPDH | Forward | GAAGGTGAAGGTCGGAGTC |
|  | Reverse | GAAGATGGTGATGGGATTTC |
| circAP2B1 | Forward | CAGCCAGCATCTTCATCACC |
|  | Reverse | GTCCTTGGAGGCAATAGTGG |
| AP2B1 | Forward | TGCCAACTCCAAGATCAAGG |
|  | Reverse | CCAGCATCATCACCAGGTTC |
| circCEP95 | Forward | TGACCAGATTCCCAAGCAGC |
|  | Reverse | GGAGTCCTTGGAGGCAATGT |
| circGIT2 | Forward | ACAGTCGGAAGCGTACTCCA |
|  | Reverse | TCCCAACTCACCAATACCGC |
| circSNX14 | Forward | GACTTCCGCAACATCAAGCA |
|  | Reverse | CGTAGTCGTCCTCGTTGTCG |
| ARG1 | Forward | CTCCAAGCCAAAGTCCTTAGAG |
|  | Reverse | AGGAGCTGTCATTAGGGACATC |
| IL10 | Forward | GCCAAGCCTTGTCAGAAAATGA |
|  | Reverse | TTTCTCAGGGGCTCGGTCAGC |
| CD163 | Forward | CACCTGTCTCAGATGCCAGG |
|  | Reverse | CACAGTGTGCCCATCTTGTG |
| CD206 | Forward | AGAAATGGAGATTTCCCTTCAGG |
|  | Reverse | TTTCTGGGTTCAGGATCACG |
| MFN2 | Forward | GCAGAGGAAACTGCTGAAGC |
|  | Reverse | ATGGTCAAGGCCACATCAGT |

| **Table S3. Antibodies used in this study** | | |
| --- | --- | --- |
| **Antibody** | **Manufacturers** | **Applications** |
| E-Cadherin | #3195, Cell Signaling Technology, Beverly, MA, USA | 1:1000 for WB  1:400 for IHC |
| Claudin-1 | #13255, Cell Signaling Technology, Beverly, MA, USA | 1:1000 for WB |
| N-Cadherin | #13116, Cell Signaling Technology, Beverly, MA, USA | 1:1000 for WB  1:100 for IHC |
| Vimentin | #5741, Cell Signaling Technology, Beverly, MA, USA | 1:1000 for WB |
| Snail | #3879, Cell Signaling Technology, Beverly, MA, USA | 1:1000 for WB |
| Slug | #9585, Cell Signaling Technology, Beverly, MA, USA | 1:1000 for WB |
| GAPDH | #2118, Cell Signaling Technology, Beverly, MA, USA | 1:1000 for WB |
| CD9 | **#**HA721533, HUABIO, Hangzhou, China | 1:2000 for WB |
| CD63 | **#**ET1607-2, HUABIO, Hangzhou, China | 1:2000 for WB |
| CD81 | **#**ET1611-87, HUABIO, Hangzhou, China | 1:2000 for WB |
| Alix | **#**ET1705-74, HUABIO, Hangzhou, China | 1:1000 for WB |
| HSP70 | **#** ET1601-11, HUABIO, Hangzhou, China | 1:2000 for WB |
| TSG101 | **#**ET1701-59, HUABIO, Hangzhou, China | 1:2000 for WB |
| Calnexin | **#**ET1611-86, HUABIO, Hangzhou, China | 1:2000 for WB |
| Histon H3 | **#** M1309-1, HUABIO, Hangzhou, China | 1:10000 for WB |
| β-actin | **#**ET1702-67, HUABIO, Hangzhou, China | 1:20000 for WB |
| Arginase-1 | **#**HA721147, HUABIO, Hangzhou, China | 1:2000 for WB |
| CD163 | **#**EM1901-90, HUABIO, Hangzhou, China | 1:100 for IF  1:1000 for WB |
| CD206 | **#**HA722892, HUABIO, Hangzhou, China | 1:100 for IF  1:1000 for WB |
| MFN2 | **#**HA720073, HUABIO, Hangzhou, China | 1:100 for IHC  1:1000 for WB |
| ESRRA | **#**ET1610-14, HUABIO, Hangzhou, China | 1:1000 for WB |
| KPNA1 | **#**ET1705-61, HUABIO, Hangzhou, China | 1:1000 for WB |
| Flag-Tag | #14793, Cell Signaling Technology, Beverly, MA, USA | 1:50 for IP  1:1000 for WB  1:400 for IF |
| Ki-67 | #ET1609-34, HUABIO, Hangzhou, China | 1:100 for IHC |
| CD68 | **#**EM1901-93, HUABIO, Hangzhou, China | 1:100 for IF |
| β-tublin | **#**ET1701-98, HUABIO, Hangzhou, China | 1:1000 for WB |
| PARP | **#**ET1608-10, HUABIO, Hangzhou, China | 1:1000 for WB |
| HRP-linked anti-rabbit IgG | #RS0002, Immunoway, CA, USA | 1:10000 for WB |
| HRP-linked anti-mouse IgG | #RS0001, Immunoway, CA, USA | 1:10000 for WB |
| Anti-mouse IgG (H+L) (Alexa Fluor® 594 Conjugate) | #ZF-0513, ZSGB-BIO, Beijing, China | 1:100 for IF |
| Anti-rabbit IgG (H+L) (Alexa Fluor® 488 Conjugate) | #ZF-0513, ZSGB-BIO, Beijing, China | 1:100 for IF |

**Supplementary Figures**

**Figure S1. (Related to Figure 1) Diagnostic and prognostic performance of serum exosomal circAP2B1.** (**A**) ROC curves of circAP2B1, CEA, and their combination for ESCC diagnosis (n = 158 per group). (**B**) PFS curves of ESCC patients with high vs. low circAP2B1 expression (n = 79 each). ***P* < 0.01.

**Figure S2. (Related to Figure 2) Single-cell RNA-seq Analysis of the ESCC Microenvironment.** (**A**) t-SNE plot showing major cell types in ESCC tissues and NATs from the GSE145370 dataset. (**B**) Expression of M2-TAM markers CD163 and CD206 across different cell clusters. (**C**) Proportion of macrophages in NATs, early-stage, and late-stage ESCC tissues from the GSE145370 dataset. (**D**) t‑SNE plot of immune cell subsets identified by scRNA‑seq in circAP2B1‑high and circAP2B1‑low ESCC tissues (n = 3 per group). (**E**) UMAP plot showing the distribution of cells from circAP2B1‑high vs. circAP2B1‑low samples. (**F**) Quantification of immune cell subset proportions. (**G**) M2 module scores of macrophages in circAP2B1‑high vs. circAP2B1‑low groups. **P* < 0.05, and ****P* < 0.001.

**Figure S3. (Related to Figure 3) Exosomal circAP2B1 is specifically secreted by ESCC epithelial cells and transferred to macrophages.** (**A**) circAP2B1 levels in exosomes from sorted epithelial, immune, and stromal cells of ESCC tissues and NATs (n=12 each). (**B**) circAP2B1 levels in KYSE30 cells and their exosomes after circAP2B1 overexpression. (**C**) circAP2B1 levels in THP‑1 macrophages co‑cultured with KYSE30 cells with or without GW4869 pretreatment. (**D**) circAP2B1 levels in THP‑1 macrophages treated with Exo‑circAP2B1 with or without GW4869 pretreatment. ****P* < 0.001.

**Figure S4. Oligomycin reverses Exo‑circAP2B1‑induced M2 polarization. (Related to Figure 4)** (**A**) Western blot analysis of M2 macrophage markers (Arginase‑1, CD163, CD206) in macrophages treated with PBS (M/PBS), M/CIRC‑Exo, M/CIRC+GW4869‑Exo, or M/CIRC-Exo+Oligomycin. GAPDH was used as a loading control. (**B**) Immunofluorescence staining of CD68 (green) and CD206 (red) in macrophages under the indicated treatments. Nuclei were counterstained with DAPI (blue). Scale bar = 50 μm. (**C**) Flow cytometry analysis of CD11b and CD206 expression in macrophages treated as indicated. Representative plots and quantification of CD11b⁺CD206⁺ M2 macrophage percentages are shown. ****P* < 0.001.

**Figure S5. (Related to Figure 5) Additional evidence for the circAP2B1/ESRRA/KPNA1 ternary complex and its nuclear translocation.** (**A**) RIP assay for circAP2B1 and ESRRA interaction in RAW264.7 macrophages. Data are representative of three independent experiments. (**B**) Domain mapping of circAP2B1 to identify the HR2 domain for ESRRA binding. Data are representative of three independent experiments. (**C**) RIP assay for circAP2B1 and KPNA1 interaction in macrophages. Data are representative of three independent experiments. (**D**) RNA FISH analysis of circAP2B1 subcellular localization in THP‑1 and RAW264.7 macrophages. Scale bar = 10 μm (**E**) Co‑IP assay assessing the effect of circAP2B1 on the ESRRA‑KPNA1 interaction. Data are representative of three independent experiments. (**F**) Nuclear‑cytoplasmic fractionation for ESRRA and KPNA1 distribution. Data are representative of three independent experiments. (**G–H**) SPR binding analysis between circAP2B1 and the preformed ESRRA/KPNA1 complex. (**I**) Control SPR binding of circAP2B1 with ESRRA alone or KPNA1 alone. (**J**) Multiplex immunofluorescence staining of CD206, ESRRA, and MFN2 in ESCC patient tumor sections. Scale bar = 20 μm. ****P* < 0.001.

**Figure S6. (Related to Figure 6) Rescue experiments demonstrate that the circAP2B1/ESRRA/KPNA1/MFN2 axis is required for circAP2B1‑induced mitochondrial functional enhancement.** (**A-B**) Cellular ATP levels (**A**) and representative TEM images (**B**) of mitochondria in macrophages transfected with the indicated constructs. (50,000×; scale bar = 200 nm). Quantitative analysis of average mitochondrial area (μm²), cristae number per mitochondrion, and aspect ratio (length/width) is shown. ***P* < 0.01, ****P* < 0.001.

**Figure S7. (Related to Figure 8) In vivo assessment of mitochondrial homeostasis and TAM polarization.** (**A**) Representative transmission electron microscopy (TEM) images of mitochondria in tumor‑associated macrophages (TAMs) isolated from subcutaneous xenografts of the indicated groups. Scale bar = 500 nm. Quantitative analysis of average mitochondrial area (μm²), cristae number per mitochondrion, and aspect ratio (length/width) is shown. (**B**) Quantification of ATP levels in TAMs from the indicated groups. (**C**) Quantification of ROS levels in TAMs from the indicated groups. (**D**) Flow cytometry analysis of mitochondrial membrane potential (JC‑1 aggregates) in TAMs from the indicated groups. (**E**) Multiplex immunofluorescence staining of CD68, CD206, and CD163 in lung metastasis sections. Scale bar = 100 μm. (**F**) Immunohistochemistry (IHC) staining of Ki‑67, MFN2, and H&E in lung metastasis sections. Scale bar = 100 μm. ****P* < 0.001.
